# Supplementary material for: Impact of red blood cell rigidity on in vivo flow dynamics and lingering in bifurcations
Source: Biophys J. 2026 Mar 20;125(8):1904–13. doi: 10.1016/j.bpj.2026.03.023 (PMC13351919; doi:10.1016/j.bpj.2026.03.023)
Supplement: Document S1. Figures S1–S11 [file mmc1.pdf]

Biophysical Journal, Volume *125*

Supplemental information

Impact of red blood cell rigidity on in vivo flow dynamics and lingering in bifurcations

Yazdan Rashidi, Felix Maurer, Selina Wrublewsky, Khadija Larhrissi, Thomas John, Frances B. Gidley, Ashley M. Toye, Lars Kaestner, Christian Wagner, Matthias W. Laschke, and Alexis Darras

Manuscript submitted to **Biophysical** *Journal*

Article

Supplementary Material: Impact of Red Blood Cell Rigidity on in vivo Flow Dynamics and Lingering in Bifurcations

Yazdan Rashidi[1,†,*], Felix Maurer[1,†], Selina Wrublewsky[2,†], Khadija Larhrissi[1,3], Thomas John[1], Frances B. Gidley[4], Ashley M. Toye[4], Lars Kaestner[1,5], Christian Wagner[1,6], Matthias W. Laschke[2,‡], and Alexis Darras[1,7,‡,*]

[1]Experimental Physics, Saarland University, Saarbrücken, Germany
[2]Institute for Clinical and Experimental Surgery, Saarland University, PharmaScienceHub (PSH), 66421 Homburg, Germany
[3]Interdisciplinary Laboratory of Physics (LIPhy), University Grenoble Alpes, 38000 Grenoble, France
[4]School of Biochemistry and Biomedical Sciences, Biomedical Sciences Building, University of Bristol, Bristol, UK
[5]Theoretical Medicine and Biosciences, Saarland University, 66421 Homburg, Germany
[6]Physics and Materials Science Research Unit, University of Luxembourg, L-1511 Luxembourg, Luxembourg
[7]School of Physics, University of Bristol, Tyndall Avenue, Bristol, BS8 1TL, United Kingdom
[*]Correspondence: yazdan.rashidi@uni-saarland.de, alexis.darras@bristol.ac.uk
[†,‡]These author contributed equally to this work.

SUPPLEMENTAL DATA

This PDF file includes: Supporting text, Supp. Fig. S1 to S11, and SI References.

SAMPLE AND ANIMAL PREPARATION

The sample and animal model (mesocricetus auratus) preparations are summarized in Supp. Fig. S1. For the injected suspension, around 2 mL whole blood was extracted from a donor animal. RBCs were separated by centrifugation and divided into two aliquots. One was treated with diamide for rigidification, the other remained untreated. The same washing procedures were used for both cell populations, but different staining protocols were used. CTDR was used for healthy cells, PKH26 for rigid cells, and FITC for plasma. Dorsal skinfold chambers were implanted in pre-weaning animals. After an adjustment time of three days, the freshly prepared stained blood sample was injected and fluorescence microscopy performed immediately afterwards.

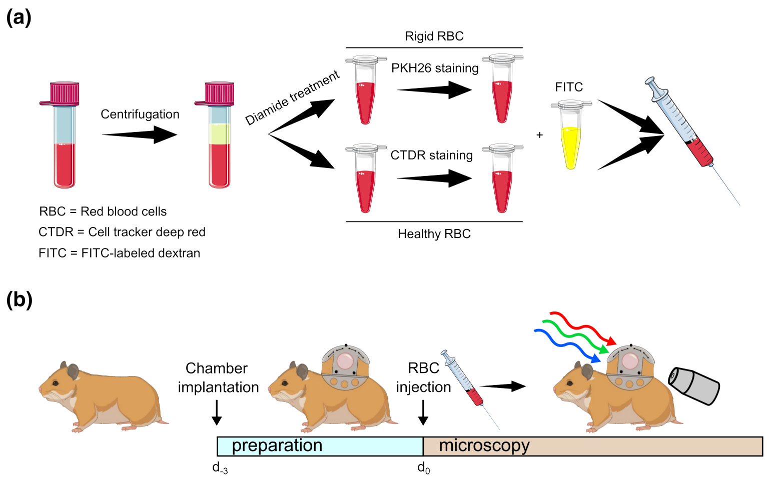

Supplemental Figure **S1**: **Preparation of blood samples and the animal model. (a)** Blood from a donor animal is extracted, RBCs separated and divided into two aliquots. One is treated with diamide for cell rigidification. The two aliquots are stained differently and suspended in FITC-labeled dextran and prepared for injection. **(b)** The skinfold chamber is surgically implanted on d_{-3}. On day of imaging d_0, the blood sample including the three dyes is injected and the chamber is imaged using fluorescence microscopy. Graphics elements adapted from (1).

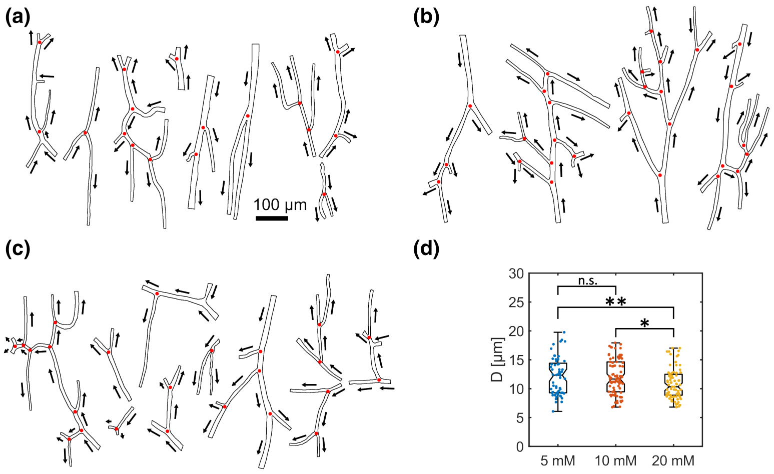

Supplemental Figure **S2**: **Comparison of datasets.** Manually traced masks of the analyzed microvascular geometries are shown for **(a)** 5 mM, **(b)** 10 mM and **(c)** 20 mM diamide. The scaling is equal in those panels. Arrows show the flow direction, red dots mark the analyzed bifurcations. Distributions of the diameter along each vessel are shown in **(d)**. The diameters of 5 mM, $\langle D \rangle = 12.1(4)$ µm, and 10 mM, $\langle D \rangle = 12.0(4)$ µm, are significantly different from the 20 mM dataset, $\langle D \rangle = 10.8(4)$ µm ($p = 0.008$ and $p = 0.013$). The diameters are not significantly different between 5 mM and 10 mM ($p = 0.89$).

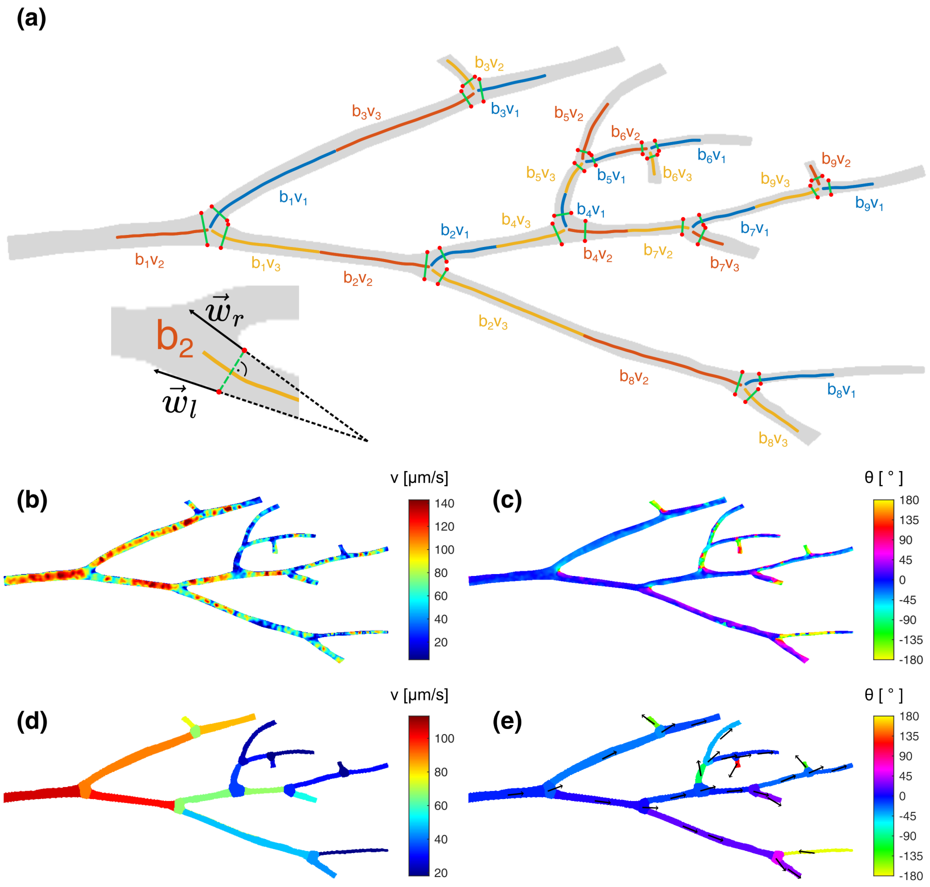

Supplemental Figure **S3**: **Geometry and bulk flow analysis.** **(a)** A mask of the channel interior was created manually, see gray shading. An automatized algorithm found vessel center lines (orange, yellow and blue) and borders of bifurcations (green with red dots). The bifurcation borders were found by the angle between tangential vessel wall vectors $\vec{w}_r$ and $\vec{w}_l$ from two points on the walls where the connection is perpendicular to the vessel center line. Each bifurcation was indexed and has three attached vessels $b_i v_j$, $i = 1, ..., 8$, $j = 1, ..., 3$. **(b)** The Lukas Kanade estimation for optical flow was applied on the footage of stained plasma to estimate the bulk flow speed. **(c)** The flow direction (θ w.r.t. x-axis) was extracted from the flow components. **(d)** Average flow in each region. **(e)** average angle in each region. All bifurcations were arterioles except b_8.

IMAGING AND IMAGE PROCESSING

Microscopic videos were recorded as sequences of $H \times 1024\,\text{px}^2$ grayscale images in uint16 format, where H (≤ 638) depends on the chosen cropped region of interest. The FITC-labeled dextran stained plasma was recorded at 250 fps, CTDR stained RBCs at 50 fps, and PKH26 stained RBCs at 100 fps due to the differing fluorescence signal intensities between stains, while using the maximum allowable exposure time. Each selected region of the vascular network was recorded first with excitation of FITC, afterwards with an alternation between CTDR and PKH26 illumination for a total of five times. In each sequence 2250 frames were recorded. The first two sequences of each cell type were not analyzed to exclude the duration of the tissue adjustment to the illumination. Experiments were carried out for different concentrations of diamide, 5 mM, 10 mM and 20 mM. Supp. Fig. S2 shows a comparison of the three datasets. The stained plasma image is a two-dimensional projection of the three-dimensional vascular network. In a first step, the average plasma image was computed. Vessel walls were identified and traced manually, resulting in a number of single or cascaded bifurcations for analysis. Image processing algorithms performed geometrical measurements on the traced vessel outlines. The numbers of analyzed bifurcations are 18 for 5 mM, 28 for 10 mM and 24 for 20 mM, see Supp. Fig. S2. The distributions of diameters are shown in Supp. Fig. S2. The diameters in the datasets for 5 mM and 10 mM are not significantly different, while both are significantly different from the 20 mM dataset. The 20 mM dataset contains a large portion of smaller capillary vessels, shorter in length and smaller in diameter. Manually drawn masks were input into a homemade algorithm to detect vessel center lines and bifurcation areas, see Supp. Fig. S3 **(a)**. This detection is

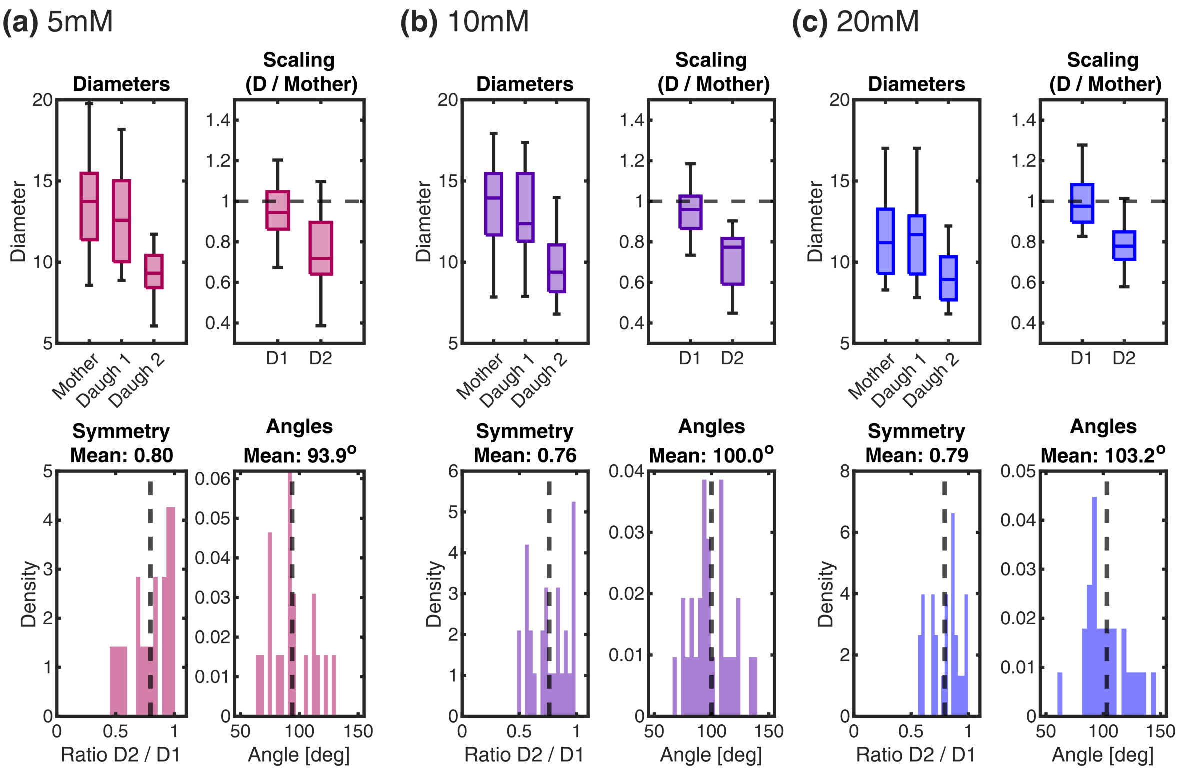

Supplemental Figure **S4**: **Geometry characteristics of the network.** Panels (a–c) display the geometric features of the analyzed network for diamide concentrations of 5 mM, 10 mM, and 20 mM, respectively. For each concentration, four panels are shown: the first panel presents the diameter of each segment at the bifurcations; the second panel shows the fractional diameters of the daughter branches relative to the mother branch, where D1 corresponds to the first daughter branch and D2 to the second; the third panel illustrates the distribution of fractional diameters of the daughter branches; and the fourth panel depicts the distribution of angles between the two daughter branches.

purely based on geometry or morphology. Center lines were detected after skeletonization. The angle of the right vessel wall $\vec{w}_r$ to the left wall $\vec{w}_l$ was used to determine the transition into the bifurcation area by the angle of the vessel walls $\sphericalangle(\vec{w}_l, \vec{w}_r) > 18°$. The apparent vessel width was then measured along the vessel. In order to distinguish between arterioles and venules, we estimated the bulk flow through the network. The Lukas-Kanade algorithm for optical flow estimation was employed to extract flow components from the fluorescence footage of stained plasma, where cells appear dark due to absorption and lack of emission. The bulk flow velocity vectors were extracted, see Supp. Fig. S3 **(b)** and **(c)**. For increased robustness, the average flow in each vessel is computed. This level of resolution was sufficient for subsequent analysis. The average flow and flow direction in each region were used for a predictive search in the cell tracking algorithm.

ADDITIONAL VELOCITY DATA

For the concentrations 5 mM and 20 mM the cell velocities in different regions of the bifurcation are presented in Supp. Fig. S6, while data for 10 mM is shown in the main text. Overall, the data show the same trends compared to 10 mM. At 5 mM compared to 10 mM, smaller differences in deceleration between healthy and rigid cells in the bifurcation area can be observed (Supp. Fig. S6 **(e)**), and no significant difference in acceleration can be observed at the beginning of the daughter branches (Supp. Fig. S6 **(f)**). This might be attributed to the lower concentration of diamide and associated lower rigidity of the rigid cell population. The 20 mM dataset shows a slightly higher speed of rigid cells in the mother branches on the 10 % level of significance, while the remaining trends are comparable to the 10 mM dataset. The higher concentration of diamide should have an equal or stronger effect on cell rigidity, increasing the differences between cell populations. However, the 20 mM dataset contains different vascular geometries compared to the 5 mM and 10 mM datasets, which share a similar set of geometries, see Supp. Fig. S2. Therefore a direct comparison of the 20 mM condition has a reduced interpretive value.

speed differences for 5 mM and 20 mM between bifurcation regions are shown in Supp. Fig. S7. For 5 mM the transitions

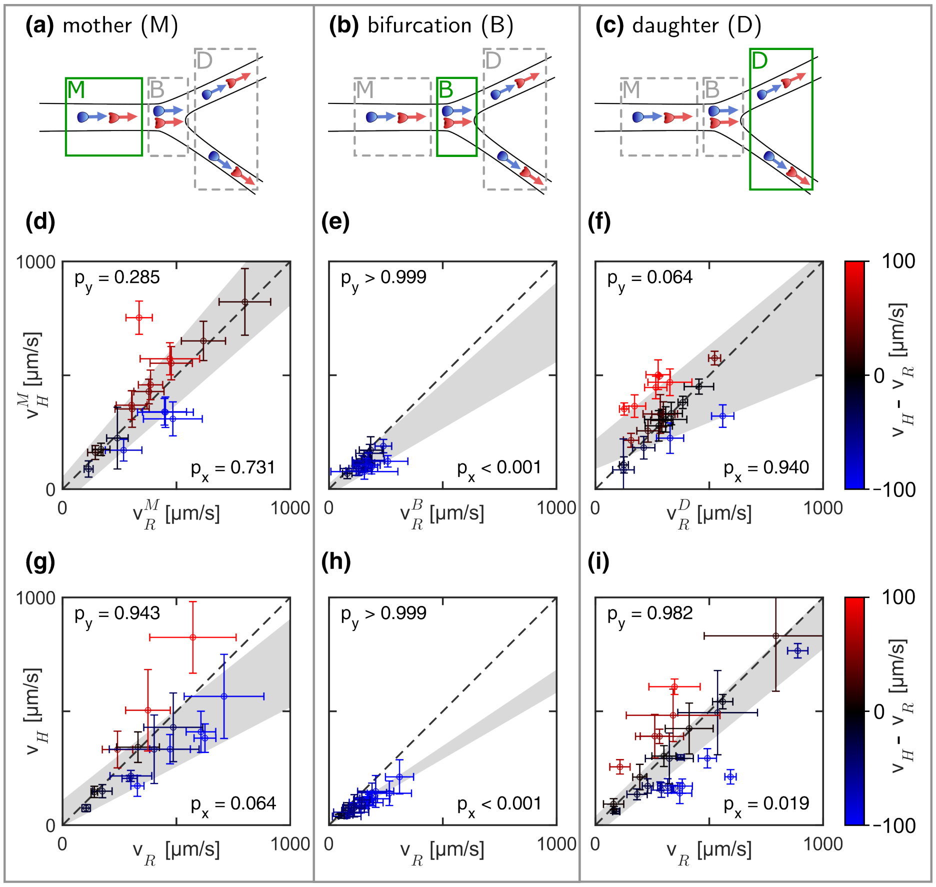

Supplemental Figure **S5**: **Comparison of mean speed for healthy vs. rigid RBCs (5 mM and 20 mM Diamide) across bifurcation regions. (a)-(c)** Schematics of a bifurcation with the ROI as mother vessel (M), bifurcation (B), or daughters (D) highlighted in green, healthy cells in red and rigid cells in blue, and below the mean velocities in each ROI respectively, with rigid RBC velocities on the x-axis and healthy RBC velocities on the y-axis. **(d)-(f)** data for 5 mM, **(g)-(i)** data for 20 mM. Panels share equal y-axes. Each data point shows data from one bifurcation. The dashed line represents equal velocities (identity line). Data points above this line, where healthy RBCs have higher velocities, are shaded increasingly red with greater distance from the line $v_H - v_R$. Points below the line, indicating higher velocities for rigid RBCs, are shaded increasingly blue, see colorbar. Error bars reflect the standard deviation of the cell population. P-values p_x and p_y denote the statistical significance from the sign rank test: $p_y < 0.05$ indicates significantly higher velocities for healthy RBCs, and $p_x < 0.05$ indicates significantly higher velocities for rigid RBCs. The gray filled area shows the 95 % confidence range of a linear regression.

from mother branch to bifurcation and bifurcation to daughter branches exhibit the same behavior as for 10 mM in the main text Fig.4, with a stronger deceleration effect for healthy cells. The 20 mM dataset does not show these clear trends. The difference from comparing average velocities in Supp. Fig. S6 is that the data in Supp. Fig. S7 is correlated, as differences from the transition of individual cells are taken. Specifically in the 20 mM dataset, the velocity data, Supp. Fig. S6 **(g)** indicates that rigid cells are slightly faster in the mother branches. A possible reason might be that vessels in the 20 mM dataset are shorter. The length might not be sufficient for a lateral migration of interacting cells back to the vessel center. We showed that the number of interacting cells is also higher for healthy cells in the 20 mM dataset (Supp. Fig. S8). There are two mechanisms that could potentially increase the fraction of interacting cells and the average lingering time for one cell type over the other. The first is a distribution closer to the apex leading to a higher number of cells that can interact with the apex. The second mechanism is a higher deformability leading to stronger deformation in case of interaction and lingering. For all datasets we can exclude the first mechanism as a reason for the observed differences between healthy and rigid cells. Supp. Fig. S8 **(a)-(c)** show the fraction of cells that can interact with the apex given by the number of trajectories with points located within 4 µm of the apex. There are no significant differences between healthy and rigid conditions for all concentrations of diamide. This means that the interaction probability for healthy and rigid cells by their spatial distribution is similar and differences in the lingering time and

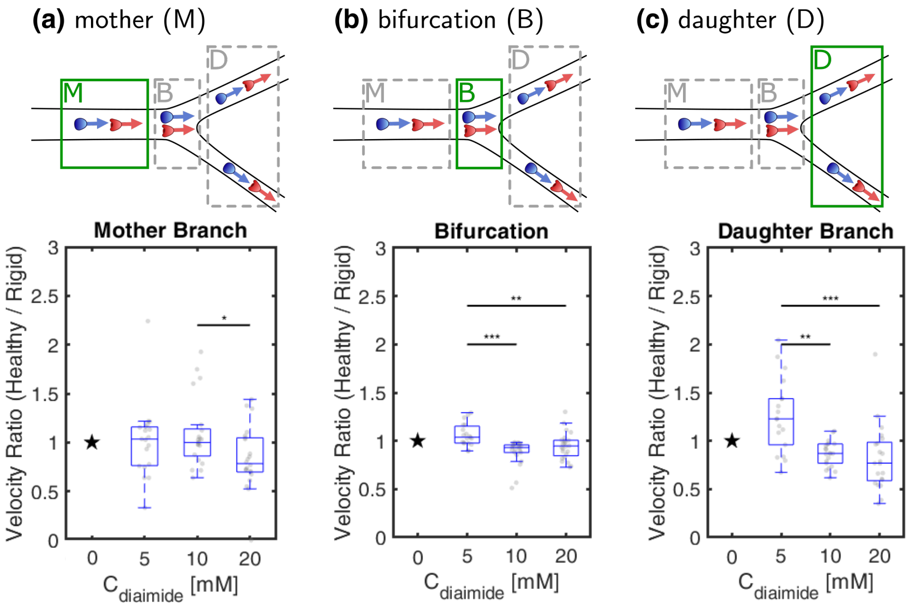

Supplemental Figure **S6**: **Velocity ratio for different diamide concentrations in each segment of the bifurcations. (a)-(c)** Schematics of a bifurcation with the ROI as mother vessel (M), bifurcation (B), or daughters (D) highlighted in green, healthy cells in red and rigid cells in blue, and below the velocities ration as a function of diamide concentration in each ROI respectively. The value at 0 mM represents the expectation, which should be equal to one, as indicated by star. Each data point shows the average for one bifurcation, and statistical significance was evaluated using a two-sample Student t-test. Significant differences are marked with a (*), indicating a p-value less than 0.05.

lateral migration can be attributed solely to differences in deformability. The lingering Peclet number is higher for healthy cells in all datasets, see Supp. Fig. S8 **(d)-(f)**.

LATERAL MIGRATION OF HEALTHY CELLS

The speed distributions of healthy and rigid cells in different regions around the bifurcation are compared in the main text, Fig. 6. In general, the variance of the speed distribution in the beginning of the daughter branch is higher than in the end. Healthy cells exhibit a sub-population of slower-moving cells in the beginning which is reduced in the end of the daughter branch. For the example bifurcation in main Fig. 6**(a)**, the total distance versus the total time spent in each region is shown in Supp. Fig. S9. The two-dimensional distributions show a similar drop shape in each region and for both cell types. There is a tail that appears only in the distribution of healthy cells in the beginning of daughter branches. It belongs to cells that move approximately the same distance as the average distance of all cells but need more time. We conclude that this fraction of cells corresponds to migrating cells interacting with the apex.

To quantify the change in velocity distribution at a bifurcation, we first define the Velocity Drop Ratio (R) for a specific cell type. Let v_M be the velocity of the cell in the mother vessel, and v_{D1} and v_{D2} be the velocities in the first and second daughter vessels, respectively. The partition asymmetry for a given cell type is calculated as the ratio of the velocity drop in the second daughter branch to the velocity drop in the first daughter branch:

$$R = \frac{|v_m - v_{D2}|}{|v_m - v_{D1}|}. \qquad (1)$$

In our experiment, we measure this ratio simultaneously for two populations within the same geometry. To determine how cell stiffness alters flow partitioning relative to the healthy baseline, we define the comparative metric δ as the ratio of the healthy drop ratio to the rigid drop ratio:

$$\delta = \frac{R_H}{R_{rigid}} = \frac{\left(\frac{|v_m - v_{D2}|}{|v_m - v_{D1}|}\right)_H}{\left(\frac{|v_m - v_{D2}|}{|v_m - v_{D1}|}\right)_{rigid}}. \qquad (2)$$

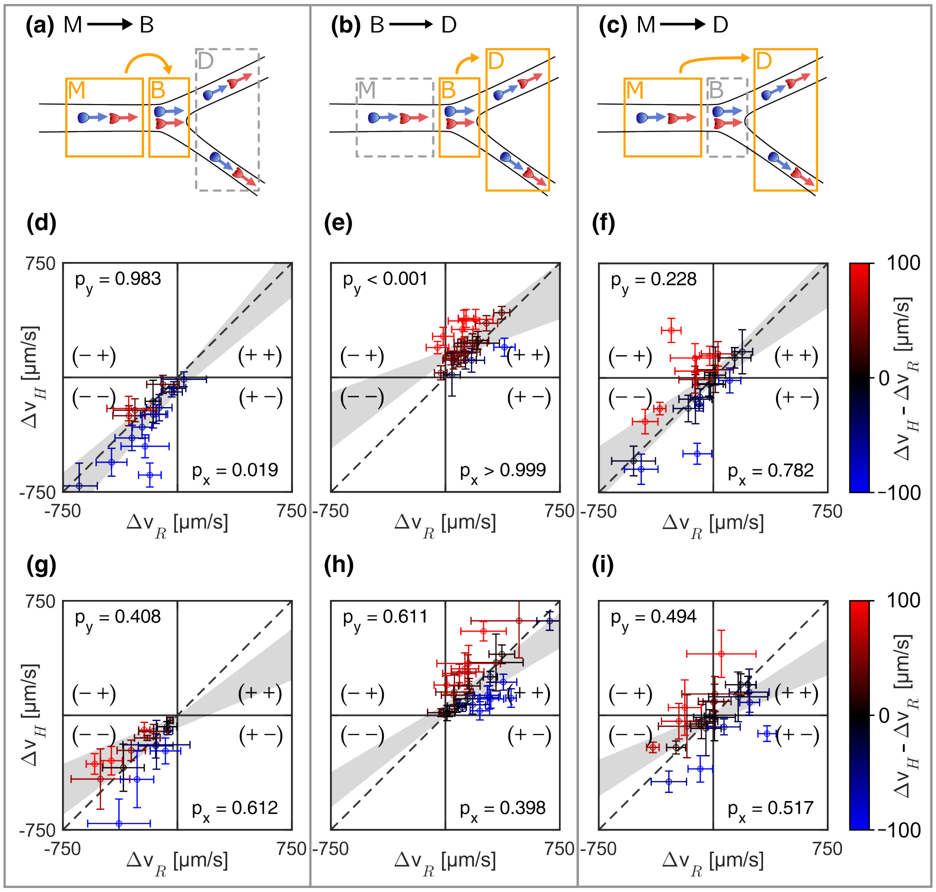

Supplemental Figure **S7**: **Comparison of change in speed for healthy vs. rigid (5 mM and 20 mM Diamide) RBCs across bifurcation regions. (a-c)** Schematics of a bifurcation with the compared regions from mother vessel (M) to bifurcation (B), bifurcation (B) to daughter (D), and mother (M) to daughter (D) highlighted in orange. **(d-f)** Changes in speed, defined as downstream speed minus upstream speed, respectively, rigid RBCs on the x-axis and for healthy RBCs on the y-axis, for 5 mM. **(g-i)** Data for 20 mM diamide concentration. Panels share equal y-axes. The dashed line represents equal velocities (identity line), while solid lines indicate zero change for rigid and healthy RBCs. The point color is according to the distance $\Delta v_H - \Delta v_R$, see colorbar. Error bars reflect statistical experimental uncertainties. A reference to each quadrant by the signs of x and y values is given by (++), (−+), (−−), and (+−). P-values p_x and p_y denote the statistical significance from the sign rank test: $p < 0.05$ indicates significantly higher differences, i.e. a stronger acceleration for (++) or deceleration for (−−). The gray filled area shows the 95 % confidence range of a linear regression.

Supp. Fig. S10 illustrates the distribution of δ across three distinct stiffness conditions (5 mM, 10 mM, and 20 mM). Statistical significance against a null hypothesis of $\delta = 1$ is indicated above each group.

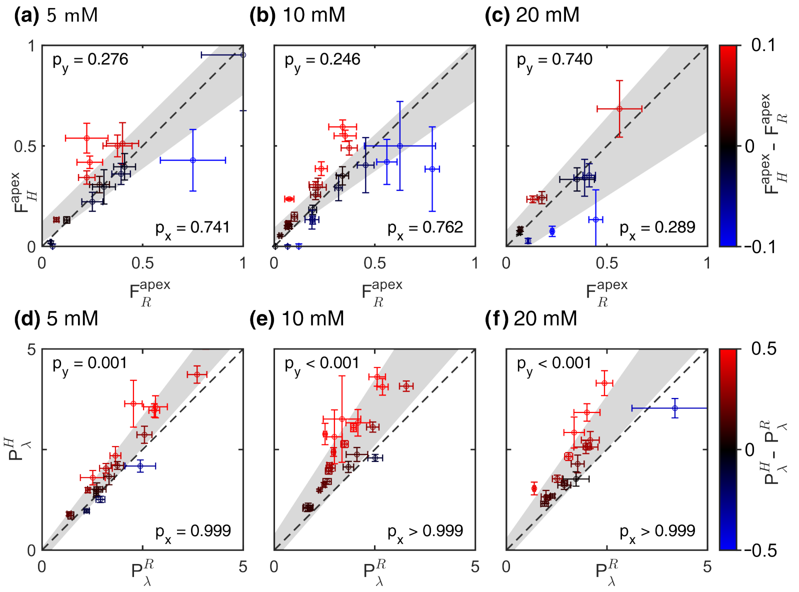

Supplemental Figure **S8**: **Fraction of cells near the apex and lingering. (a-c)** The axes' variables F^{apex} are the number of trajectories with points closer than 4 µm to the apex of the bifurcation divided by the total number of trajectories. Each panel shows the data for a different diamide concentration, **(a)** 5 mM, **(b)** 10 mM, **(c)** 20 mM. **(d-f)** The lingering Peclet number P_λ is the residence time normalized by the advection time. It is significantly higher for healthy cells in all datasets, **(d)** 5 mM, **(e)** 10 mM and **(f)** 20 mM.

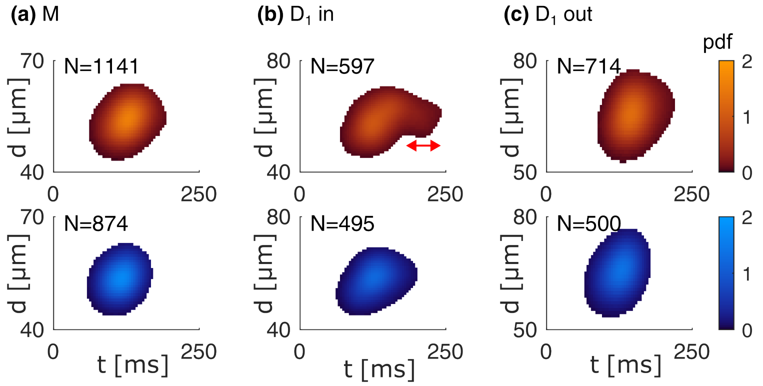

Supplemental Figure **S9**: **Distance versus time in bifurcation regions. Two-dimensional distributions.** Diagrams show the probability density by kernel density estimation of the total distance and the total time spent in **(a)** the end of the mother, **(b)** the beginning of the daughter, and **(c)** the end of the daughter branch. Data for healthy cells in the top row is colored in red, for rigid cells in blue, and the number of detected trajectories *N* is provided. The red double arrow indicates a distribution tail only found for healthy cells in the beginning of the daughter branch. At a similar distance, the tail expands approximately 50 ms in time.

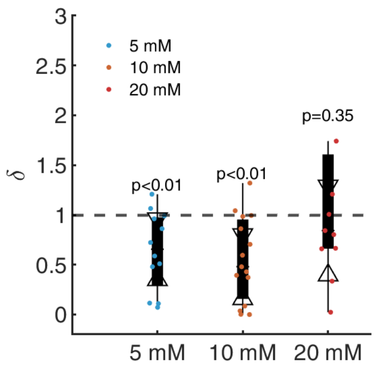

Supplemental Figure **S10**: **Comparison of Flow Asymmetry** (δ)**.** Boxplots showing the ratio of velocity drops for varying stiffening conditions. The horizontal dashed line at $\delta = 1$ represents zero deviation from healthy behavior. (n varies per condition; P-values calculated via t-test against 1).

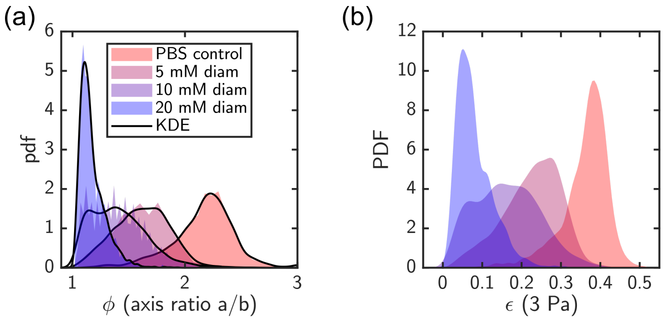

Supplemental Figure **S11**: **Distributions of RBCs elongation under well-controlled shear stresses. (a)** Measurements of the elongation, **(b)** associated deformation.

REFERENCES

1. BioRender, 2024. BioRender. https://biorender.com. Accessed: 2025-05-15.
