# Supplementary material for: Impact of red blood cell rigidity on in vivo flow dynamics and lingering in bifurcations
Source: Biophys J. 2026 Mar 20;125(8):1904–13. doi: 10.1016/j.bpj.2026.03.023 (PMC13351919; doi:10.1016/j.bpj.2026.03.023)
Supplement: Document S2. Article plus supporting material [file mmc3.pdf]

Biophysical Journal
Article

Biophysical Society

Impact of red blood cell rigidity on in vivo flow dynamics and lingering in bifurcations

Yazdan Rashidi,[1,8,*] Felix Maurer,[1,8] Selina Wrublewsky,[2,8] Khadija Larhrissi,[1,3] Thomas John,[1] Frances B. Gidley,[4] Ashley M. Toye,[4] Lars Kaestner,[1,5] Christian Wagner,[1,6] Matthias W. Laschke,[2,9] and Alexis Darras[1,7,9,*]

[1]Experimental Physics, Saarland University, Saarbrücken, Germany; [2]Institute for Clinical and Experimental Surgery, Saarland University, PharmaScienceHub (PSH), Homburg, Germany; [3]Interdisciplinary Laboratory of Physics (LIPhy), University Grenoble Alpes, 38000 Grenoble, France; [4]School of Biochemistry and Biomedical Sciences, Biomedical Sciences Building, University of Bristol, Bristol, UK; [5]Theoretical Medicine and Biosciences, Saarland University, Homburg, Germany; [6]Physics and Materials Science Research Unit, University of Luxembourg, Luxembourg, Luxembourg; and [7]School of Physics, University of Bristol, Bristol, UK
[8]These authors contributed equally
[9]These authors contributed equally

ABSTRACT Bifurcations are a crucial part of the mammalian microvasculature, as they establish the interface between blood and tissue. The flexibility of red blood cells (RBCs), the main cellular constituent of blood, is believed to strongly impact their partitioning, quantitative in vivo measurements have so far been elusive. This study investigates the effect of cell rigidity on the lateral movement after arteriole bifurcations and lingering by comparing the movement of artificially rigidified RBCs with that of healthy RBCs in vivo. Lingering describes a recently highlighted phenomenon in which RBCs reside in the bifurcation between two branches before entering either one. Our results show that increased RBC rigidity reduces lingering and, contrary to expectations, leaves healthy RBCs with a lower speed than rigidified RBCs in some areas of the microcirculation. We conclude that rigid RBCs show a different flow behavior leading to reduced wall contact potentially altering endothelial signaling and nutrient delivery and show a different partitioning at bifurcations.

SIGNIFICANCE Red blood cells (RBCs) are highly deformable, enabling their efficient passage through the microvasculature. This study reveals, for the first time in vivo, that RBC rigidity significantly reduces lingering, a transient slowing and residence at capillary bifurcations, thereby altering their downstream partitioning. Contrary to prevailing assumptions, rigidified RBCs do not universally impair flow. Instead, their average transit time is shorter than that of healthy cells in certain segments. These findings reshape our understanding of how cell deformability influences microcirculatory dynamics and challenge simplified views of rigid RBC behavior in blood flow. This work has broad implications for diseases characterized by altered RBC rigidity, such as malaria or sickle cell disease, and informs therapeutic strategies targeting microvascular function.

INTRODUCTION

The distribution of red blood cells (RBCs) within the complex network of the microcirculation serves as a critical determinant in the delivery of oxygen to tissues. This vital process depends on the complex interplay between the spatial arrangement of microvessels and the behavior of RBCs as they transit through them (1–6). Single-cell flow through bifurcations is crucial, as it precedes and determines capillary flow. The distribution of RBCs through a bifurcation is significantly influenced by the geometry of the bifurcation apex (3,4,7,8). Previous findings show that the daughter vessel with a higher flow rate tends to collect a relatively higher number of RBCs compared with the vessel with a lower flow rate (3,7,8). Pioneering works highlighted the role of the flow velocity, bifurcation size, and blood viscosity on the phase separation, for vessel radii spanning from 20 to 100μm (1,9,10). However, recent studies, in silico, in vitro, and in vivo, revealed significant deviations from traditional empirical models (8,11–17). A previously unexpected phenomenon emerged from these recent

Submitted September 19, 2025, and accepted for publication March 10, 2026.

*Correspondence: yazdan.rashidi@uni-saarland.de or alexis.darras@bristol.ac.uk

Editor: Karin Leiderman.

https://doi.org/10.1016/j.bpj.2026.03.023

© 2026 The Author(s). Published by Elsevier Inc. on behalf of Biophysical Society.
This is an open access article under the CC BY license (http://creativecommons.org/licenses/by/4.0/).

works: RBCs tend to linger at the apex of bifurcations due to possible interactions with the bifurcation apex (11,16). This means that these cells temporarily reside near the apex of the bifurcation, where their speed diminishes, changing the flow dynamics. Consequently, this behavior impacts the distribution of cells entering downstream daughter vessels and changes the characteristic distances between cells, creating intermittent voids (11,16,18,19). The rigidity of RBCs is commonly believed to impair blood flow and influence the spatial and temporal organization of flowing RBCs. Diseases such as malaria, diabetes, sickle cell disease, and acanthocytosis often feature RBCs with impaired deformability (4,20–24). Rigid RBCs significantly alter the viscosity and shear-thinning characteristics of blood, consequently impacting hemorheology, flow resistance, and microvascular perfusion (25–28). Furthermore, RBC deformability plays a crucial role in shaping the emergence of a cell-free layer (CFL) within complex microvascular geometries (24,29). More accurately, in blood vessels with a luminal diameter bigger than a few cell diameters, RBCs tend to move toward the central axis of the vessel. This lateral migration leads to the development of a CFL near the vessel walls and a higher concentration of RBCs in the central region of the flow. The lift forces generated by shear stress gradients in the Poiseuille flow profile are the main mechanism driving RBCs to the vessel center (30). A parabolic speed distribution is typical of laminar flow within these microvessels. The maximum fluid speed occurs at the center, thus RBCs located closer to the center are carried away more rapidly. This phenomenon leads to an increase in the hematocrit level of the blood discharged from the vessel, when compared with the tube hematocrit, a well-known mechanism termed the Fahraeus effect, and it also reduces the apparent viscosity of blood due to the presence of the CFL known as the Fahraeus-Lindqvist effect (30–32). These hemodynamic effects and margination have been employed in microfluidic applications for plasma separation extraction and white blood cell isolation from whole blood samples (33–37). These applications rely on the significant differences in deformability and size between the targeted cells and RBCs to perform efficient separation. The biomimetic separation principle can be extended to differentiate between normal and malaria-infected RBCs (38). Unlike in earlier studies, where targeted cells exhibited substantial differences from RBCs in both size and stiffness, malaria-infected RBCs differ from healthy RBCs primarily in deformability while maintaining similar sizes to healthy RBCs. Despite this understanding, the underlying mechanisms by which rigid RBCs alter blood flow remain largely uncharacterized.

In this study, we explore how the deformability of RBCs affects their transit through bifurcations, including their lingering time. In previous investigations, where we showcased the influence of lingering on RBC partitioning in capillary bifurcations within the microcirculation, we found that an increase in the lingering Péclet number corresponds to a greater deviation from the Zweifach-Fung empirical model, demonstrating a direct correlation (11,18). Cheng et al.(39) demonstrated that, unlike healthy RBCs which migrate toward the vessel center, sickle RBCs exhibit a distinct margination behavior, migrating toward the vessel wall. These differences in lateral migration cause sickle cells to preferentially enter lower flow daughter branches, a reversal of typical RBCs partitioning at bifurcations, which may reduce their likelihood of lingering since sickle RBCs are positioned toward the walls. This work reveals that healthy cells are more likely to experience deceleration at bifurcations and, statistically, have a slower average speed than rigidified cells at the entrance of daughter vessels, challenging the widely accepted idea that more rigid cells simply impair blood flow. While this might be the case in longer vessels, the redistribution of the cells after a bifurcation complicates the situation, as healthy cells flow slower locally in the network. Further, the most pronounced differences in spatial distribution between healthy and rigid cells occur near the entrances of the daughter branches; overall, downstream migration of healthy cells within the daughter vessels appears sufficient to homogenize the distributions from the proximal to the distal segments.

MATERIALS AND METHODS

In vivo experiments

Permissions

The experiments were conducted according to the German legislation on animal protection, the ARRIVE guidelines, the European legislation on the protection of animals (Directive 2010/63/EU) and the NIH Guidelines on the Care and Use of Laboratory Animals (NIH publication no. 85-23 Rev. 1985), and received approval from the local authorities (State Office for Consumer Protection, Saarbrücken, Germany; permission no. 25/2018).

Animal preparation and microscopy. The hamsters were kept under a standard 12/12-h day/night cycle, with access to water and food ad libitum. Hamsters with an age of 5 to 7 weeks, weighing 55 to 70 g, were used for the implantation of a dorsal skinfold chamber (40). The surgery was performed under deep anesthesia, using 150 mg/kg ketamin (Serumwerke Bernburg, Bernburg, Germany) and 0.25 mg/kg domitor (Orion Pharma, Espoo, Finland) intraperitoneally, with intraoperative pain medication by carprofen (5 mg/kg, Zoetis, Hagen, Germany) subcutaneously. Briefly, the back of the hamsters was shaved and a titanium chamber consisting of two frames was implanted on the lifted dorsal skinfold as shown in Fig. 1 *a* and *b*. In the area of circular observation window (diameter of 10 mm), the cutis, subcutis, and retractor muscles were removed to expose the striated skin muscle for later observation of the microcirculation. The window was closed with a cover glass that was fixed with a snap ring. Animals were allowed to recover for 72 h after the procedure.

Hamsters were anesthetized as described above prior to intravital microscopy. Healthy and rigid RBCs from a donor hamster (see materials and methods, rigidification of RBCs) were resuspended with fluorescent plasma marker fluorescein isothiocyanate (FITC)-labeled dextran (5%, 150 kDa (Sigma-Aldrich, Taufkirchen, Germany), and were injected retro-orbitally. The animal was fixed on a plexiglass stage during microscopy as shown in Fig. 1 *c*. Several capillary bifurcations in different areas of the chamber window were observed using epifluorescence microscopy (Axio Examiner

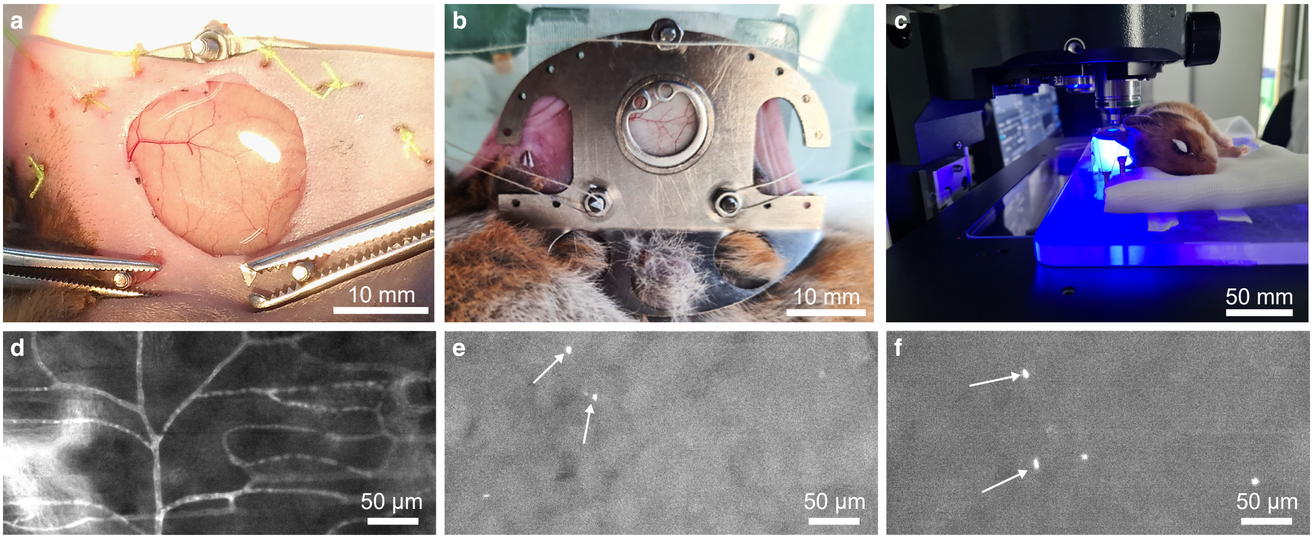

FIGURE 1 Hamster dorsal skinfold chamber model and in vivo imaging: (*a*) the cutis, subcutis, and retractor muscles were removed to expose the striated skin muscle, facilitating subsequent observation of the microcirculation. (*b*) A dorsal skinfold chamber was implanted on the back of a hamster. (*c*) The anesthetized hamster was positioned beneath the objective of an epifluorescence microscope. (*d*) Example of a microvascular network imaged by fluorescence microscopy with an LED illumination central filter wavelength of 469 nm. The FITC-stained plasma appears bright. (*e*) Healthy RBCs were dyed with CTDR, and observed by fluorescence with an illumination central filter wavelength of 631 nm. Arrows indicate individual cells. (*f*) Fluorescence image obtained at 555 nm, showing bright rigid cells stained with PKH26.

A1, Zeiss, Oberkochen, Germany). FITC-labeled dextran was excited with a peak wavelength 469 nm LED (Colibri 7, Zeiss). For fluorescence images, see Fig. 1 *d*. Imaging was performed with 20× (LD A-Plan, NA = 0.35, Zeiss), 50× (LD EC Epiplan-Neofluar, NA = 0.55, Zeiss) or 100× (LD C Epiplan-Neofluar 100×, NA = 0.75, Zeiss) long-distance objectives. Video acquisition was carried out with a digital camera (Orca Flash 4.0, C13440, Hamamatsu Photonics, Hamamatsu, Japan) using the software ZEN 3.1 Blue (Zeiss).

Rigidification and fluorescent labeling of RBCs. Blood was collected from the vena cava of a hamster and centrifuged to separate the RBCs from the plasma. Aliquots of 100 μL from the RBC pellets were placed into separate Eppendorf tubes. The RBCs were then incubated with varying diamide (Sigma-Aldrich) concentrations (5, 10, and 20 mM). Each concentration was prepared by diluting diamide in 4 mL of phosphate-buffered saline (PBS) solution (Gibco, Thermo Fisher Scientific, Schwerte, Germany), followed by a 30-min incubation at room temperature with gentle rotation. After the incubation period, the cells were centrifuged for 5 min at 900 rcf to create a pellet, and the supernatant was removed carefully. For fluorescent labeling, different staining protocols were used for healthy and rigid RBCs. Healthy RBCs were stained using CellTracker ™ (Invitrogen, Thermo Fisher Scientific) Deep Red (CTDR). Example microscopy images of stained cells are shown in Fig. 1 *e*. The cells were incubated with 2 μL CTDR in 1 mL PBS at 37 °C for 30 min. Rigidified RBCs were stained with PKH26 (Sigma-Aldrich) dye. For fluorescence images, see Fig. 1 *f*. Specifically, 400 μL of diluent C (Sigma-Aldrich) was added to and mixed with the RBC pellet. The sample was transferred to a 50 mL tube. Separately, 500 μL of diluent C was mixed with 5 μL PKH26 and vortexed. This PKH26 mixture was added to the RBC suspension, mixed thoroughly, and incubated for 5 min. After staining, the cells were washed with 20 mL PBS containing 1 − 2 % bovine serum albumin (Sigma-Aldrich) and centrifuged for 6 min at 900 rcf. The supernatant was carefully removed after centrifugation. Before injection, the stained RBC pellets were resuspended in FITC-labeled dextran, and retro-orbitally injected into the animals. The distribution and speed of rigid cells observed in this study, by virtue of being performed in vivo, do take into account physiological flows within large microvascular beds (cells are injected retro-orbitally and observations are performed in the skinfold chamber). We are therefore not restricted to small systems, although our observations were performed only in arterioles and we could not report on the thinnest capillaries, as it was not possible to detect a statistically significant number of cells in each of them within a reasonable observation time. For a summary of the preparation also refer to the supporting material, sample and animal preparation, and Fig. S1.

Effect of diamide on red blood cell elongation in shear and in flow

Automated rheoscope techniques (41) have made it possible to rapidly measure full distributions of RBCs elongation under well-controlled shear stresses in vitro. Here, we combine such rheoscope measurements of diamide-stiffened RBCs with our estimates of the shear stress distribution in microvessels. For each analyzed vessel segment in the in vivo movies, we estimate the local wall shear stress τ_w from the measured diameter and RBC velocity. Across all vessels and diamide conditions (5, 10, and 20 mM), the resulting shear stresses lie in the sub-Pa to few-Pa range, with a tail extending to around 3 Pa. Within this broad distribution, the data set-wide mean wall shear stress is approximately $\langle \tau_w \rangle \approx 0.5\,\text{Pa}$. In the rheoscope experiment, RBCs are sheared in Couette flow and imaged at a fixed shear stress τ (41). For each cell, the in-plane projected shape is approximated by an ellipse with major axis a and minor axis b, and the axis ratio is defined as[(Eq. 1)]

$$\phi = \frac{a}{b} \geq 1. \qquad \text{(Eq. 1)}$$

Following standard practice in RBC rheology (41), we use the deformation index[(Eq. 2)]

$$\epsilon = \frac{a-b}{a+b} = \frac{\phi - 1}{\phi + 1}, \qquad \text{(Eq. 2)}$$

which ranges from $\epsilon = 0$ for a circle ($\phi = 1$) to $\epsilon \to 1$ for extremely elongated cells. The rheoscope, therefore, provides a full empirical probability density $p_\phi(\phi \mid \tau)$ under each chemical condition and at each applied shear stress. The rheoscope software exported the measured distributions as discrete PDF values for ϕ (or an equivalent elongation measure) at 3 Pa under different concentrations of diamide: PBS control, 5, 10, and 20 mM diamide. The corresponding data are shown in Fig. S11. To propagate these distributions through our deformation and lingering calculations in a way that is straightforward to visualize, we pseudo-sample from the discrete PDFs. For each pseudo-sampled axis ratio ϕ_i, we compute the corresponding ϵ_i, providing empirical distributions $p_\epsilon(\epsilon \mid \tau = 3\,\text{Pa})$ for each condition. From the pseudo-sampled distributions, we obtain the mean and standard deviation of ϕ and ϵ at 3 Pa. The key statistics used in the remainder of the analysis are summarized in Table 1.

Image analysis and tracking algorithm

The first step of image processing involved detecting the vascular network features and the bulk fluid flow. Each network geometry was masked out manually by finding the apparent vessel walls in the intensity averaged plasma fluorescent image. The mask covers a single bifurcation or a sequence of bifurcations. The geometrical features were extracted from the mask using a custom algorithm. The bulk flow was estimated from the plasma footage and used in cell tracking for a predictive search. For more information, see the supporting material, imaging and image processing, and Figs. S2 and S3. For cell tracking, a spatiotemporal filtering of the intensity signal was performed. The CZI file format by Zeiss was read in MATLAB using the Bioformats library (42). The encoder saved the gray scale frames in 16 bit unsigned integer format. In vivo footage is prone to flickering. Hence, the intensity histogram of each frame was measured and normalized. For cell detection, an intensity outlier map was computed, i.e., the normalized intensity ratio (NIR) of each pixel (i,j) in frame k.(Eq. 3)

$$\text{NIR}(i,j,k) = \frac{|V(i,j,k) - \langle V(i,j,k)\rangle_k|}{\sigma_k(V(i,j,k))}, \qquad \text{(Eq. 3)}$$

where $V(i,j,k)$ is the intensity value, $\langle . \rangle_k$ is the average over the time dimension, $\sigma_k(.)$ the corresponding standard deviation. Statistical temporal outliers, such as moving single cells, cause high NIR values. Noise also contributes to outliers in time statistics. To distinguish cells from noise, a spatial correlation was calculated. This involves a median filter of a size corresponding to the cell radius R and a convolution with a structure array of $2R$. The cell diameter for Syrian hamsters was reported to be 7µm (43), and good filtering results were achieved in tests with this filter size. Local maxima in the resulting map exceeding an empirical noise threshold were counted as cell detection events. An example frame processing is shown in Fig. 2 *a* and *b*. The entirety of all N events in one video make up a point cloud in space-time (x_l, y_l, ut_l), $l = 1, \ldots, N$, where the x-y-plane is the imaging plane, see Fig. 2 *c*. The characteristic velocity u is chosen to equal the approximate average cell velocity magnitude. Consequently, successive points belonging to the same trajectory might be found in a spherical search volume. The radius was chosen by eye to optimize the separation and yield of trajectories. This analysis focuses on individual vessels labeled by a hand drawn mask. Points are singled out and grouped accordingly. Each point cloud was denoised (44). An initial point clustering by minimal Euclidean distance was followed by a spline regression refining filter. A spline was fitted to each cluster using shape language modeling (45). Scattered points were dismissed, leaving valid cell trajectories. After parameterization by pathlength, each trajectory was sampled with the same pathlength increment through interpolation. Fig 2 *d* depicts the resulting filtered trajectories. The velocity magnitude of each point was estimated using forward differences.

TABLE 1 Axis ratio ϕ and deformation index ϵ from rheoscope measurements under controlled shear at 3 Pa

Condition	$\phi(3\,\text{Pa})$	$\epsilon(3\,\text{Pa})$
PBS control	2.16 ± 0.27	0.36 ± 0.06
5 mM diamide	1.61 ± 0.25	0.23 ± 0.07
10 mM diamide	1.41 ± 0.25	0.16 ± 0.08
20 mM diamide	1.18 ± 0.12	0.081 ± 0.047

Means and standard deviations are computed from pseudo-samples drawn from the empirical PDFs.

Lingering quantification

In this study, flow and cell statistics were characterized within time intervals typically ranging from 20 to 50 s. Quasisteady flow conditions were ensured by looking at times short enough that the average flow rate did not significantly change. However, the chosen measurement time was long enough to observe a significant number of cells, between 10 and 1,000, passing through the vessel (46). By selecting suitable time intervals, we were able to define flow and cell statistics consistently. Recent studies highlighting RBC lingering as an important influence on microvascular flow introduced several methods to quantify the lingering of RBCs (11,18,47). The latest method, which demonstrated higher robustness (47), defines a relative residence time τ_{RBC} as the normalized time that an RBC spends within the bifurcation region. This is calculated as:(Eq. 4)

$$\tau_{RBC} = \frac{t_{r,RBC}}{t_{ref}}, \qquad \text{(Eq. 4)}$$

where $t_{r,RBC}$ represents the actual residence time of a specific RBC in the bifurcation region, and t_{ref} is a reference time. The latter is defined as:(Eq. 5)

$$t_{ref} = \frac{L}{u_m}, \qquad \text{(Eq. 5)}$$

Here, L is the minimum length from the end of the mother vessel to the bifurcation apex. The mean speed in the second half of the mother vessel is u_m. This reference time is a convective timescale for RBC transport through the bifurcation region. The average dimensionless lingering time $P_\lambda = \langle \tau_{\text{RBC}} \rangle$, representing the average time each cell spends passing through the bifurcation, is also referred to as the lingering Péclet number, defined as the ratio of the average lingering time to the characteristic advection time.

The signed-rank test

This test was chosen because it is independent of a normalization in contrast to methods like the population t test. The nonparametric statistical method developed by Wilcoxon evaluates whether the median of paired differences in a dataset is significantly different from zero (48,49). We compare the same quantity from two different conditions assigned to the x and y axis, respectively. Data points on the identity line implicate no difference between the conditions. The signed-rank test calculates the distance of each data point to the identity line. Then the absolute distances are sorted, i.e., ranked. A sum of the sign of all distances weighted by the rank provides a measure for the imbalance toward one side of the identity line. The calculated p value is the probability that an imbalance appeared only by chance, assuming a binomial distribution of the signs of deviations. If the p value falls below a predetermined significance level (in our case 0.05), the null hypothesis of the data points being centered around the identity line is rejected, suggesting a significant difference. Conversely, a p value exceeding the significance level fails to reject the null hypothesis.

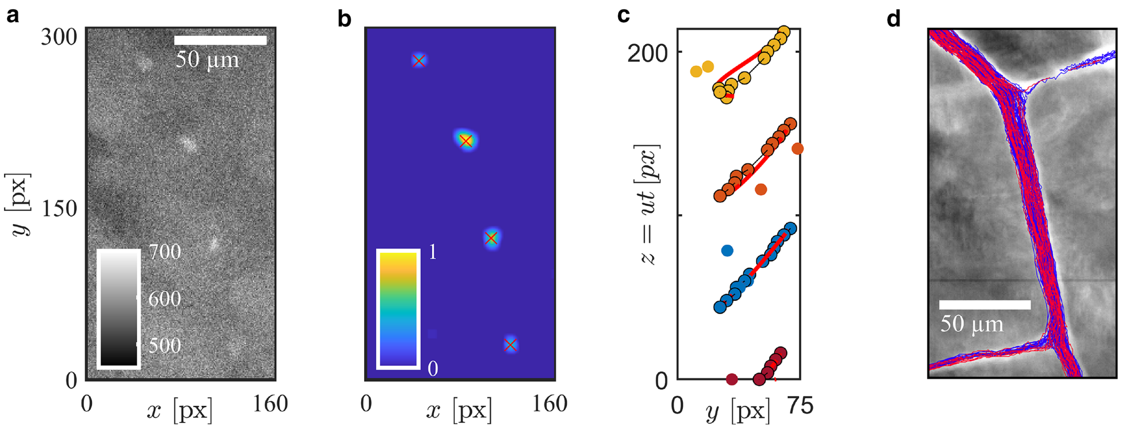

FIGURE 2 Image processing and tracking algorithm: (*a*) crop of unfiltered camera frame with static background, statistical noise, flickering, and low-contrast cell signals. (*b*) Processed NIR map, red crosses mark local maxima, i.e., detection events. (*c*) Spline filtering step: point cloud representation of all detection events including non-cell detections. Projection on the y-z plane. Different clusters are shown in different colors. Red lines represent fitted 3D splines. Points distant to splines are dismissed as noise. The remaining trajectory points are marked by a black edge and connected. (*d*) Final trajectories of healthy (red) and rigid (blue) cells drawn on stained plasma image.

RESULTS AND DISCUSSION

Velocity analysis of RBCs in bifurcations

The investigation of the effect of diamide on the deformability of RBCs, as outlined in Nouaman et al., (50), revealed that diamide induces rigidity in RBCs, consequently limiting their deformability. We were able to detect and track the maximum intensity positions of RBCs with sufficient accuracy. This enabled us to understand how individual cells move within the vessels, including how they concentrate in certain areas. The statistical analysis of velocities and residence times provides insights into the effect of rigidity on RBC behavior.

In our analysis of bifurcations, we defined three distinct regions by the flow direction: the mother branch (M), the bifurcation area (B), and the daughter branches (D) (see Fig. 3). For the definition of the bifurcation borders, separating the three vessels, see supporting material and Fig. S3. We tracked the trajectories of individual cells through these regions and calculated the average speed for each area (M, B, and D) from the distribution of velocities of all locally passing RBCs with sufficient trajectory length of $>70\%$ the vessel length. It is well established that the speed of RBCs is higher in the center of the blood vessel and decreases toward the vessel walls. Fig. 3 shows exemplary data for healthy and rigidified cells. Statistical differences between paired data points were evaluated by the signed-rank test for deviations from the identity line, where values for healthy and rigid cells are equal. While there is no difference in mother branches Fig. 3 *a*, healthy cells are significantly slower in the bifurcation area, see Fig. 3 *b*. This shift might occur because healthy RBCs, flowing

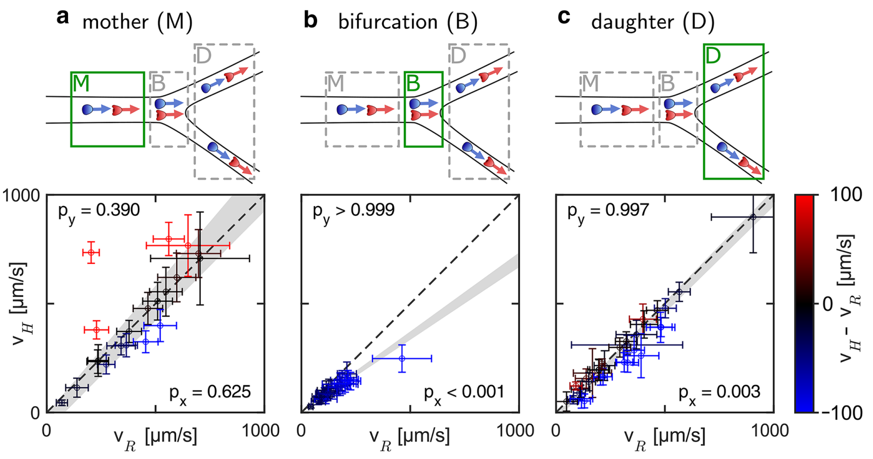

FIGURE 3 Comparison of mean speed for healthy versus rigid (10 mM diamide) RBCs across bifurcation regions. (*a–c*) Schematics of a bifurcation with the ROI as mother vessel (M), bifurcation (B), and daughters (D) highlighted in green, healthy cells in red, and rigid cells in blue. Below, the mean velocities in each ROI, respectively, with rigid RBC velocities on the *x* axis and healthy RBC velocities on the *y* axis. Panels share equal *y* axes. Each point shows data from one bifurcation, data for both daughter branches in (*c*). The dashed line represents equal velocities (identity line). Data points above this line, where healthy RBCs have higher velocities, are shaded increasingly red with greater distance from the line. Points below the line, indicating lower velocities for healthy RBCs, are shaded increasingly blue. Error bars reflect the standard deviation of the cell population. *p* values p_x and p_y denote the statistical significance from the signed-rank test: $p_y < 0.05$ indicates significantly higher velocities for healthy RBCs, and $p_x < 0.05$ indicates significantly lower velocities for healthy RBCs. The gray filled area shows the 95% confidence range of a linear regression.

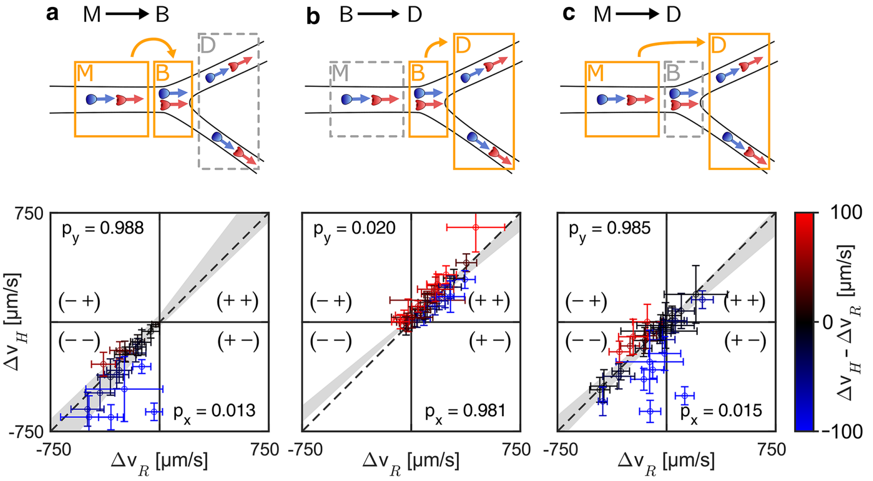

FIGURE 4 Comparison of change in speed for healthy versus rigid (10 mM diamide) RBCs across bifurcation regions. (*a–c*) Schematics of a bifurcation with the compared regions from mother vessel (M) to bifurcation (B), bifurcation (B) to daughter (D), and mother (M) to daughter (D) highlighted in orange, and below the changes in speed, defined as downstream speed minus upstream speed, respectively, with the change in speed for rigid RBCs on the x axis and for healthy RBCs on the y axis. Panels share equal y axes. The dashed line represents equal velocities (identity line), while solid lines indicate zero change for rigid and healthy RBCs. Error bars reflect statistical experimental uncertainties. A reference to each quadrant by the signs of x and y values is given by $(+\,+)$, $(-\ \ +)$, $(-\ \ -)$, and $(+\ \ -)$. p values p_x and p_y denote the statistical significance from the signed-rank test: $p < 0.05$ indicates significantly higher differences, i.e., a stronger acceleration for $(+\,+)$ or deceleration for $(-\ \ -)$. The gray filled area shows the 95% confidence range of a linear regression. The deceleration from (M) to (B) is in most bifurcations maintained comparing (D) to (M) despite the acceleration from (B) to (D). There is a stronger net deceleration for healthy cells across a bifurcation.

near the centerline in the mother vessel tend to follow streamlines toward the central stagnation point. However, we found no significant difference in the number of cells near the apex between healthy and rigid condition, see Fig. S8. While some bifurcations show a higher fraction of healthy, and others a higher fraction of rigid cells near the apex, for almost all bifurcations in all diamide conditions, the lingering time was significantly higher for healthy cells. The interaction seems to depend on cell properties or speed rather than spatial distribution in the bifurcation. This indicates that the differences in speed originate in the deformability determined interaction with the vessel wall at the apex rather than the lateral distribution of cells. In the mother branches, the speed is not significantly different between healthy and rigid cells. This implies that any differences in and directly after the bifurcation are neutralized during the flow along the early section of the branching vessels. However, the difference in speed in the bifurcation transfers into the daughter vessels (Fig. 3 *c*). In theory, healthy RBCs tend to linger longer at the apex of the bifurcation due to their interaction with the apex. As a result, they deform significantly and slide along the vessel walls. This process takes time, causing the RBCs to gradually migrate back to the center of the vessel in the subsequent daughter branches. This different behavior aligns with the results in Fig. 3 *c*, where healthy RBCs display lower average velocities than rigid RBCs in the daughter branches. Although the average velocity of healthy RBCs is lower, our imaging approach does not allow cell-type-specific flux measurements, and velocity alone does not determine effective perfusion or oxygen delivery given vessel branching, capillary occlusion, and reduced wall interaction of rigid cells. The results for lower (5 mM) and higher (20 mM) diamide concentrations are shown in Figs. S5 and S6. For all concentrations, the deceleration in bifurcations follows the same trend, while the differences in daughter branches are not significant at 5 mM. This can be attributed to the lower rigidity.

Since we observed distinct speed trends for RBCs in different vessel regions, we investigated the downstream differences in speed from one region to the next. Fig. 4 shows the change in speed from mother to bifurcation, bifurcation to daughter, and mother to daughter vessels. Each panel is divided into four quadrants, labeled as follows and described by the meaning of points located in each:

- $(+\,+)$: increase in speed for both rigid and healthy RBCs.
- $(-\ \ +)$: decrease in speed for rigid and increase for healthy RBCs.
- $(-\ \ -)$: decrease in speed for both rigid and healthy RBCs.
- $(+\ \ -)$: increase in speed for rigid and decrease for healthy RBCs.

In Fig. 4 *a*, the speed change from the mother vessel to the bifurcation shows that both RBC types generally experience a reduction, with all data in $(-\ \ -)$. The reduction is more pronounced for healthy RBCs, as more data points are below the identity line. In Fig. 4 *b*, the speed change from the bifurcation to the daughter vessels shows that both RBC types generally experience an increase in speed, with most data points located in $(+\,+)$. The difference between daughter and mother branches are shown in Fig. 4 *c*, where negative values indicate higher speed in the mother vessel, and positive values indicate higher speed in the daughter. The majority of data points are located in $(-\ \ -)$, indicating a decrease in speed for both populations. This agrees with the nature of the vascular network where flow decreases with advanced branching, supporting the idea that RBCs have a lower speed in smaller vessels. The decrease is more pronounced for healthy cells demonstrating that healthy RBCs tend to reduce their speed in the bifurcation,

suggesting that they linger longer in the bifurcation region compared with rigid RBCs. This behavior likely arises from the deformability effects on lingering. In some cases, the speed increases for both cell populations. Data for other concentrations of diamide is shown in Fig. S7. The slow-down and acceleration effects were significantly higher for healthy cells in the 5 and 10 mM, not in the 20 mM data. The geometry in the 20 mM dataset is not directly comparable due to the high portion of capillaries of smaller diameter (see Figs. S2 and S4). A smaller diameter introduces stronger confinement effects which can slow rigid cells down, leveling interaction effects for healthy and resistance effects for rigid cells.

To identify lingering RBCs for each bifurcation, we followed the method described by Bucciarelli et al. (47). The parameter $P_\lambda = \langle \tau_{RBC} \rangle$ represents the average relative lingering time for all stained RBCs passing through the bifurcation.

Fig 5 shows results of the lingering analysis. The example trajectories in Fig. 5 *a* show that those cells with a higher residence time in the bifurcation are located closer to the apex and are more likely to interact with the wall. A comparison of the lingering Péclet number between rigid and healthy cells is shown in Fig. 5 *b*. For almost all bifurcations, the lingering Péclet number is higher in case of healthy cells. That means the ratio of Péclet numbers is greater than one. A comparison for different diamide concentrations is shown in Fig. 5 *c*, where the *y* axis represents the ratio of the lingering time of healthy cells to that of rigid cells, with each point corresponding to a specific bifurcation. In the figure, the 0 mM concentration of diamide serves as a theoretical prediction, where no difference is expected between RBC types, resulting in a lingering ratio of one. As shown in Fig. 5 *c*, for all diamide concentrations, the lingering ratio exceeds one, indicating that healthy cells linger longer than rigid cells. This outcome could be attributed to two factors: 1) healthy cells are more deformable than rigid cells and can deform due to the interaction with the apex of a bifurcation, causing them to remain longer in the bifurcation; 2) differences in margination forces on healthy and rigid cells in the bifurcation lead to pronounced lateral migration of healthy cells.

However, as there is no significant difference in the number of cells near the apex between healthy and rigid condition, (see Fig. S8) the differences in lingering Péclet number originate in the interaction of cells with the apex rather than the lateral distribution of cells. We expect two possible explanations for why healthy RBCs remain longer in the bifurcation region compared with rigid RBCs: 1) deformation may increase the cell-wall contact area and therefore the resistance and 2) deformation may shift the cell's center of mass toward the bifurcation apex, where the flow velocity is lower. However, our raw data do not allow us to directly quantify these two effects.

To assess the significance of the difference between the lingering ratio and the predicted ratio of 1, we applied a Student's *t* test, with statistically significant increases below the 5% level indicated by () in the figure. This analysis shows a significant increase in the lingering ratio for all diamide concentrations compared with the theoretical value. There is no significant difference between the 10 and 20 mM diamide concentrations, likely because, at these levels, the cells have already reached sufficient rigidity, resulting in minimal difference between the two concentrations. The tendency toward a lower ratio for 20 mM can be attributed to the difference in recorded geometries (see supporting material). Due to limitations in our imaging method, hematocrit cannot be quantified with sufficient accuracy, and therefore we cannot perform a systematic analysis of hematocrit-dependent effects within the present data set.

Lateral migration after the bifurcation

While to this point we investigated bifurcations independent of each other, the physiological microvascular network is characterized by a sequence of bifurcations, i.e., the daughter branch of one bifurcation is also a mother branch of another downstream bifurcation. To investigate the influence of the succession of bifurcations on the cell motion, we studied connected daughter becoming mother branches. Specifically, the previously described lower speed in the inlet of daughter

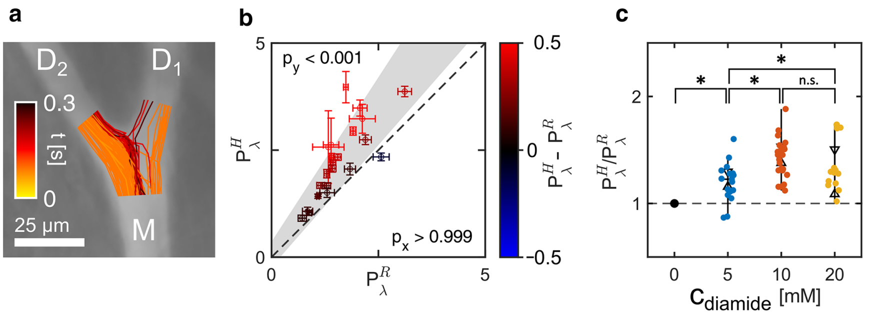

FIGURE 5 For a Figure360 author presentation of Fig. 5, see https://doi.org/10.1016/j.bpj.2026.03.023. Ratio of the lingering Péclet number of healthy to rigid RBCs. (*a*) Example bifurcation with trajectories of healthy RBCs during transition from the mother branch (M) to either one of the daughter branches, (D_1) or (D_2). The color of each line represents the time spent in the bifurcation area. (*b*) Comparison of Péclet numbers for healthy (P^H_λ) and rigid cells (P^R_λ). The error bars show the SEM. (*c*) Péclet number ratio for different diamide concentrations $c_{diamide}$. The value at 0 mM represents the expectation, which should be equal to one, as indicated by the dashed line. Each data point shows the average for one bifurcation, and statistical significance was evaluated using a two-sample Student's *t* test. Significant differences are marked with a (*), indicating a *p* value less than 0.05. The label "n.s." (not significant) indicates $p > 0.1$.

Figure360▷

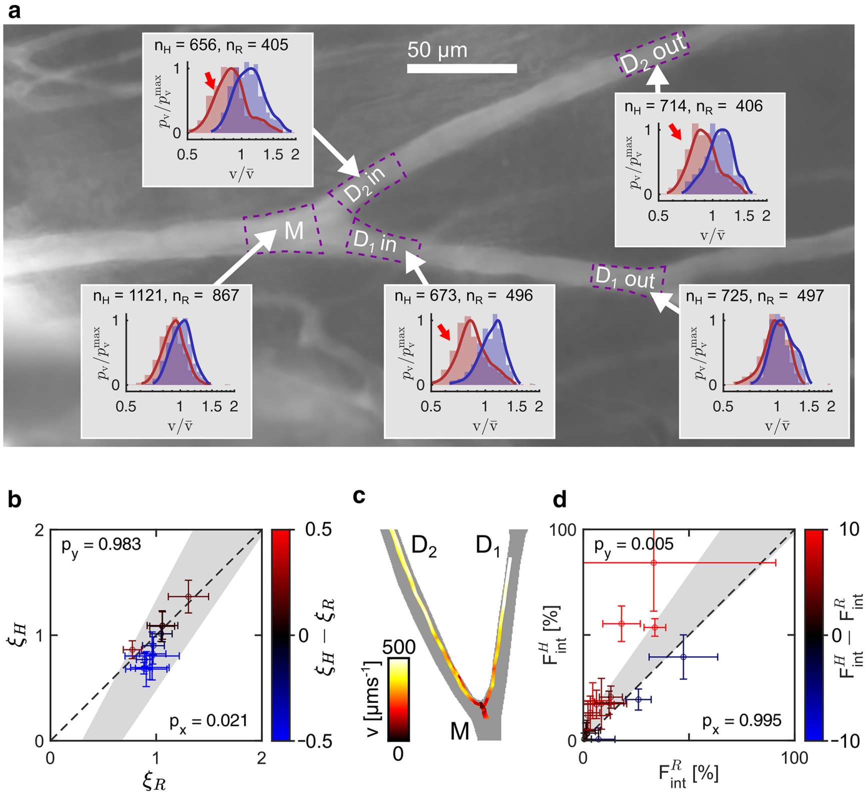

Figure360▷

FIGURE 6 For a Figure360 author presentation of Fig. 6, see https://doi.org/10.1016/j.bpj.2026.03.023. Movement of cells after the bifurcation. (*a*) Example arteriole geometry with two cascaded bifurcations. Each daughter branch is divided into two regions, in and out. Each region has the same length of 30μm and ends at the bifurcation border. Diagrams show the kernel density estimated probability density function of the speed $p_v(\mathrm{v})$ as a function of the speed, normalized by the average $\bar{\mathrm{v}} = (\mathrm{med}(\mathrm{v}_H) + \mathrm{med}(\mathrm{v}_R))/2$, in red for healthy and in blue for rigid cells. The x axis is logarithmic to show differences in lower velocities. Fractions with slower cells are marked by red arrows. Distributions in the inlet of the main D_1 $(\langle d \rangle = 13.9(2)\mu\mathrm{m}, \langle v \rangle = 344(5)\mu\mathrm{m}s^{-1})$ and inlet as well as outlet of the secondary daughter D_2 $(\langle d \rangle = 14.5(1)\mu\mathrm{m}, \langle v \rangle = 319(2)\mu\mathrm{m}s^{-1})$ are wider for healthy cells. In the end of the mother M $(\langle d \rangle = 17.6(1)\mu\mathrm{m}, \langle v \rangle = 432(3)\mu\mathrm{m}s^{-1})$. From inlet to outlet of D_1, a narrowing can be observed. (*b*) Comparison of standard deviation ratios $\xi = \sigma(p_\mathrm{v}^{\mathrm{out}})/\sigma(p_\mathrm{v}^{\mathrm{in}})$. Each point reflects data from one connecting branch. Points are shaded by the distance from the dashed identity line with blue toward the x axis and red toward the y axis. Error bars show the statistical uncertainty. Ratios below 1 can be associated with cell focusing and values below the identity line indicate a stronger effect for healthy cells. p values p_x and p_y denote the statistical significance from the signed-rank test: $p < 0.05$ indicates significantly higher focusing. The gray filled area shows the 95% confidence range of a linear regression. (*c*) Example of healthy RBCs trajectories of the first bifurcation from (*a*) with a significant increase in speed after the bifurcation, i.e., from lingering cells. Lines are color coded by the speed along each trajectory from black to yellow. (*d*) Comparison of the fraction of wall-interacting cells $\mathrm{F}_{\mathrm{int}}$ between healthy and rigid conditions. A cell counts as interacting with the vessel wall at the apex if its speed is below 30% of the average speed in branches, excluding the bifurcation area, for more than 80 ms.

branches for healthy cells and no difference in the outlet of mother branches (Fig. 3) indicates the lateral migration of interacting healthy RBCs from the wall to the center. Fig. 6 *a* shows an example of two cascaded bifurcations, where the lateral migration of healthy RBCs after interaction with the apex from the wall to the center in the daughter vessels impacts the speed distribution. We acknowledge that interpreting cell-wall interactions based on lateral migration estimated from RBC speed must be done cautiously: although velocity correlates with wall distance, our imaging configuration does not allow reliable extraction of absolute lateral positions due to small out-of-plane tissue movements and the lack of full 3D information, so we restrict ourselves to population-level trends and use velocity only as an indirect indicator of lateral positioning. The traveled distance d and the spent time t of each cell was measured for the mother branch and the first half of daughter branches belonging to the first bifurcation, as well as the second half of the daughter branch leading to the second bifurcation. In each region, the probability density function of the speed $p_v(v)$ was estimated using kernel density estimation with a Gaussian kernel. While rigid and healthy cells cross the same distance in a region, the time needed for this distance can vary. In the end of the mother branch as well as the end of the connecting daughter branch, the standard deviation $\sigma(p_\mathrm{v})$ is similar for healthy and rigid cells. However, in the beginning of the daughter branches, the healthy RBCs distribution is broader. This indicates a subpopulation of cells spending more time closer to the wall. This subpopulation exists in the inlet of the connecting daughter branch, however not in the outlet, indicating lateral migration toward the center. Trajectory distances versus time duration data is discussed in Fig. S9. Fig. 6 *b* shows a systematic comparison of the width ratio of outlet to inlet for 10 mM diamide concentration between healthy and rigid cells for various connected vessels in the dataset. A ratio lower than one shows focusing. The ratio is close to one for rigid cells leading to the conclusion that rigid cells do not experience focusing. On the other hand, the ratio for healthy cells is in most cases below one. This result suggests that interactions in the bifurcation lead to a subpopulation of healthy cells that migrate laterally in the daughter branch to the center, and this effect is suppressed by rigidification. Furthermore, the data suggest that, for the studied geometries, the vessel length is sufficient for migration to take an effect on cell partitioning.

To identify and separate the migrating subpopulation, we defined a trajectory as interacting if its speed is below 30% of the average speed in bifurcation branches for a time frame

of at least 80 ms. Fig. 6 *c* shows example trajectories classified as interacting. Those trajectories contain points in close proximity to the apex. Fig. 6 *d* shows a comparison of the fraction of interacting cells F_{int} between healthy and rigid cell populations for various bifurcations. The fraction of interacting healthy cells is with $22\% \pm 5\%$ significantly higher compared with rigid with $13\% \pm 3\%$. Those values provide an estimate for the average amount of lingering cells among the population for the investigated geometries.

CONCLUSION

Our study demonstrates, for the first time in vivo, that RBC rigidity affects lingering, i.e., RBCs slowing down and temporarily residing near the bifurcation apex, constituting an effective mechanism that significantly modifies the partitioning of cells through capillary bifurcations, Although we cannot quantify the exact 3D positions, the relative differences observed between conditions are robust with respect to potential out-of-plane bias. Interestingly, our data disproves the idea that rigidified cells have an overall increased margination in vivo, as velocities in mother branches and the second half of daughter vessels are not significantly different. These results also challenge the view that rigid cells overall impair the blood flow, as flexible cells are actually slower than rigidified cells in some parts of the network. The influence of rigidified cells in the blood flow is therefore a more nuanced picture than portrayed in most discussions.

DATA AND CODE AVAILABILITY

The data supporting this study's findings are available in Zenodo: https://doi.org/10.5281/zenodo.16812693. The Zenodo record contains the associated OneDrive link.

ACKNOWLEDGMENTS

This work was supported by the research unit FOR 2688 Wa1336/12 and LA2682/9-1 of the German Research Foundation. A.D. acknowledges funding by the Young Investigator Grant of the Saarland University. We thank Johannes G.G. Dobbe and Geert J. Streekstra, Biomedical Engineering and Physics, University of Amsterdam, Meibergdreef 9, Amsterdam, the Netherlands, for facilitating access to ARCA equipment and software for the Toye laboratory.

AUTHOR CONTRIBUTIONS

A.D., C.W., M.W.L., and L.K. designed the research. L.K., S.W., and M.W.L. designed, applied, and obtained authorizations for animal experiments. S.W. and M.W.L. performed the surgery. S.W., K.L., and M.W.L. recorded the videos. F.B.G. and A.M.T. performed the ARCA diamide measurements. Y.R. designed and implemented statistical analysis and lingering quantification methods. F.M. performed image processing and tracking algorithms. F.M.,Y.R., and K.L. refined the code. A.D. and T.J. gave theoretical input to development and employed methods. Y.R., F.M., and A.D. wrote the manuscript. All authors discussed the results and critically reviewed the article.

DECLARATION OF INTERESTS

The authors declare no competing interests.

SUPPORTING MATERIAL

Supporting material can be found online at https://doi.org/10.1016/j.bpj.2026.03.023.

REFERENCES

1. Pries, A. R., and T. W. Secomb. 2008. Blood flow in microvascular networks. *In* Microcirculation. R. F. Tuma, W. N. Duran, and K. Ley, eds Elsevier, pp. 3–36. https://doi.org/10.1016/B978-0-12-374530-9.00001-2.
2. Secomb, T. W. 2017. Blood flow in the microcirculation. *Annu. Rev. Fluid Mech.* 49:443–461. https://doi.org/10.1146/annurev-fluid-010816-060302.
3. Svanes, K., and B. W. Zweifach. 1968. Variations in small blood vessel hematocrits produced in hypothermic rats by micro-occlusion. *Microvasc. Res.* 1:210–220. https://doi.org/10.1016/0026-2862(68)90019-8.
4. Fung, Y. C., and B. W. Zweifach. 1971. Microcirculation: mechanics of blood flow in capillaries. *Annu. Rev. Fluid Mech.* 3:189–210. https://doi.org/10.1146/annurev.fl.03.010171.001201.
5. Schmid-Schönbein, G. W., R. Skalak, ..., S. Chien. 1980. Cell distribution in capillary networks. *Microvasc. Res.* 19:18–44. https://doi.org/10.1016/0026-2862(80)90082-5.
6. Li, G., T. Ye, ..., X. Li. 2023. Temporal-spatial heterogeneity of hematocrit in microvascular networks. *Phys. Fluids.* 35:021906.
7. Fung, Y.-C. 1973. Stochastic flow in capillary blood vessels. *Microvasc. Res.* 5:34–48. https://doi.org/10.1016/S0026-2862(73)80005-6.
8. Li, G., T. Ye, ..., Z. Zhu. 2023. Analysis and prediction of hematocrit in microvascular networks. *Int. J. Eng. Sci.* 191:103901.
9. Pries, A. R., K. Ley, ..., P. Gaehtgens. 1989. Red cell distribution at microvascular bifurcations. *Microvasc. Res.* 38:81–101. https://doi.org/10.1016/0026-2862(89)90018-6.
10. Fenton, B. M., R. T. Carr, and G. R. Cokelet. 1985. Nonuniform red cell distribution in 20 to 100 \mum bifurcations. *Microvasc. Res.* 29:103–126. https://doi.org/10.1016/0026-2862(85)90010-X.
11. Rashidi, Y., G. Simionato, ..., A. Darras. 2023. Red blood cell lingering modulates hematocrit distribution in the microcirculation. *Biophys. J.* 122:1526–1537. https://doi.org/10.1016/j.bpj.2023.03.020.
12. Sherwood, J. M., D. Holmes, ..., S. Balabani. 2014. Spatial distributions of red blood cells significantly alter local haemodynamics. *PLoS One.* 9:e100473. https://doi.org/10.1371/journal.pone.0100473.
13. Shen, Z., G. Coupier, ..., T. Podgorski. 2016. Inversion of hematocrit partition at microfluidic bifurcations. *Microvasc. Res.* 105:40–46. https://doi.org/10.1016/j.mvr.2015.12.009.
14. Clavica, F., A. Homsy, ..., D. Obrist. 2016. Red blood cell phase separation in symmetric and asymmetric microchannel networks: effect of capillary dilation and inflow velocity. *Sci. Rep.* 6:36763. https://doi.org/10.1038/srep36763.
15. Mantegazza, A., F. Clavica, and D. Obrist. 2020. In vitro investigations of red blood cell phase separation in a complex microchannel network. *Biomicrofluidics.* 14:014101. https://doi.org/10.1063/1.5127840.
16. Balogh, P., and P. Bagchi. 2017. Direct numerical simulation of cellular-scale blood flow in 3D microvascular networks. *Biophys. J.* 113:2815–2826. https://doi.org/10.1016/j.bpj.2017.10.020.
17. Zhou, Q., J. Fidalgo, ..., T. Krüger. 2021. Emergent cell-free layer asymmetry and biased haematocrit partition in a biomimetic vascular network of successive bifurcations. *Soft Matter.* 17:3619–3633. https://doi.org/10.1039/D0SM01845G.

18. Kihm, A., S. Quint, …, C. Wagner. 2021. Lingering dynamics in microvascular blood flow. *Biophys. J.* 120:432–439. https://doi.org/10.1016/j.bpj.2020.12.012.

19. Li, G., T. Ye, and X. Li. 2020. Parallel modeling of cell suspension flow in complex micro-networks with inflow/outflow boundary conditions. *J. Comput. Phys.* 401:109031.

20. Stuart, J., and G. B. Nash. 1990. Red cell deformability and haematological disorders. *Blood Rev.* 4:141–147. https://doi.org/10.1016/0268-960X(90)90041-P.

21. Dondorp, A. M., M. Nyanoti, …, K. Marsh. 2002. The role of reduced red cell deformability in the pathogenesis of severe falciparum malaria and its restoration by blood transfusion. *Trans. R. Soc. Trop. Med. Hyg.* 96:282–286. https://doi.org/10.1016/S0035-9203(02)90100-8.

22. Mannino, R., D. R. Myers, …, W. Lam. 2012. Increased erythrocyte rigidity is sufficient to cause endothelial dysfunction in sickle cell disease. *Blood.* 120:818. https://doi.org/10.1182/blood.V120.21.818.818.

23. Reichel, F., M. Kräter, …, J. Guck. 2022. Changes in blood cell deformability in chorea-acanthocytosis and effects of treatment with dasatinib or lithium. *Front. Physiol.* 13:852946. https://doi.org/10.3389/fphys.2022.852946.

24. Rashidi, Y., O. Aouane, …, S. M. Recktenwald. 2023. Cell-free layer development and spatial organization of healthy and rigid red blood cells in a microfluidic bifurcation. *Soft Matter.* 19:6255–6266. https://doi.org/10.1039/D3SM00517H.

25. Chien, S., S. Usami, …, M. I. Gregersen. 1967. Blood viscosity: influence of erythrocyte deformation. *Science.* 157:827–829. https://doi.org/10.1126/science.157.3790.827.

26. Passos, A., J. M. Sherwood, …, S. Balabani. 2019. The effect of deformability on the microscale flow behavior of red blood cell suspensions. *Physics of fluids.* 31:091903. https://doi.org/10.1063/1.5111189.

27. Ye, T., L. Peng, and G. Li. 2019. Red blood cell distribution in a microvascular network with successive bifurcations. *Biomech. Model. Mechanobiol.* 18:1821–1835.

28. Ye, T., X. Zhang, …, S. Wang. 2020. Biomechanics in thrombus formation from direct cellular simulations. *Phys. Rev. E.* 102:042410.

29. Abay, A., S. M. Recktenwald, …, C. Wagner. 2020. Cross-sectional focusing of red blood cells in a constricted microfluidic channel. *Soft Matter.* 16:534–543. https://doi.org/10.1039/C9SM01740B.

30. Goldsmith, H. L., G. R. Cokelet, and P. Gaehtgens. 1989. Robin Fahraeus: evolution of his concepts in cardiovascular physiology. *Am. J. Physiol.* 257:H1005. https://doi.org/10.1152/ajpheart.1989.257.3.H1005.

31. Fåhræus, R., and T. Lindqvist. 1931. The viscosity of the blood in narrow capillary tubes. *American Journal of Physiology-Legacy Content.* 96:562–568. https://doi.org/10.1152/ajplegacy.1931.96.3.562.

32. Barbee, J. H., and G. R. Cokelet. 1971. The fahraeus effect. *Microvasc. Res.* 3:6–16. https://doi.org/10.1016/0026-2862(71)90002-1.

33. Fan, R., O. Vermesh, …, J. R. Heath. 2008. Integrated barcode chips for rapid, multiplexed analysis of proteins in microliter quantities of blood. *Nat. Biotechnol.* 26:1373–1378. https://doi.org/10.1038/nbt.1507.

34. Jäggi, R. D., R. Sandoz, and C. S. Effenhauser. 2007. Microfluidic depletion of red blood cells from whole blood in high-aspect-ratio microchannels. *Microfluid. Nanofluidics.* 3:47–53. https://doi.org/10.1007/s10404-006-0104-9.

35. Shevkoplyas, S. S., T. Yoshida, …, M. W. Bitensky. 2005. Biomimetic design of a microfluidic device for auto-separation of leukocytes from whole blood. *Anal. Chem.* 77:933–937. https://doi.org/10.1021/ac049037i.

36. Lima, R., T. Ishikawa, …, T. Yamaguchi. 2008. Radial dispersion of red blood cells in blood flowing through glass capillaries: the role of hematocrit and geometry. *J. Biomech.* 41:2188–2196. https://doi.org/10.1016/j.jbiomech.2008.04.033.

37. Katanov, D., G. Gompper, and D. A. Fedosov. 2015. Microvascular blood flow resistance: role of red blood cell migration and dispersion. *Microvasc. Res.* 99:57–66. https://doi.org/10.1016/j.mvr.2015.02.006.

38. Hou, H. W., A. A. S. Bhagat, …, C. T. Lim. 2010. Deformability based cell margination—a simple microfluidic design for malaria-infected erythrocyte separation. *Lab Chip.* 10:2605–2613. https://doi.org/10.1039/C003873C.

39. Cheng, X., C. Caruso, …, M. D. Graham. 2025. Red blood cell partitioning and segregation through vascular bifurcations in a model of sickle cell disease. *Soft Matter.* 21:5793–5803. https://doi.org/10.1039/D4SM01519C.

40. Laschke, M. W., B. Vollmar, and M. D. Menger. 2011. The dorsal skinfold chamber: window into the dynamic interaction of biomaterials with their surrounding host tissue. *Eur. Cell. Mater.* 22:147–167. https://doi.org/10.22203/ecm.v022a12.

41. Dobbe, J. G. G., G. J. Streekstra, …, C. A. Grimbergen. 2002. Measurement of the distribution of red blood cell deformability using an automated rheoscope. *Cytometry.* 50:313–325.

42. Linkert, M., C. T. Rueden, …, J. R. Swedlow. 2010. Metadata Matters: Access to Image Data in the Real World. *J. Cell Biol.* 189:777–782. https://doi.org/10.1083/jcb.201004104.

43. Stewart, M. O., L. Florio, and E. R. Mugrage. 1945. Hematological findings in the golden hamster (*Cricetus auratus*). *J. Lab. Clin. Med.* 30:693–698.

44. Rusu, R. B., Z. C. Marton, …, M. Beetz. 2008. Towards 3D Point Cloud Based Object Maps for Household Environments. *Robot. Autonom. Syst.* 56:927–941. https://doi.org/10.1016/j.robot.2008.08.005.

45. D'Errico, J. 2024. SLM - Shape Language Modeling, MATLAB Central File Exchange. https://www.mathworks.com/matlabcentral/fileexchange/24443-slm-shape-language-modeling.

46. Alonzo, M., N. J. Karst, …, G. Coupier. 2024. Spatio-temporal instabilities of blood flow in a model capillary network. *Phys. Rev. Fluids.* 9:104401. https://doi.org/10.1103/PhysRevFluids.9.104401.

47. Bucciarelli, A., A. Mantegazza, …, D. Obrist. 2024. Relation between hematocrit partitioning and red blood cell lingering in a microfluidic network. *Biophys. J.* 123:3355–3365. https://doi.org/10.1016/j.bpj.2024.07.042.

48. Baguley, T. 2018. Serious Stat: A Guide to Advanced Statistics for the Behavioral Sciences. Bloomsbury publishing.

49. Shorack, G. R., and J. A. Wellner. 2009. Empirical Processes with Applications to Statistics. SIAM. https://doi.org/10.1137/1.9780898719017.bm.

50. Nouaman, M., A. Darras, …, S. M. Recktenwald. 2023. Effect of cell age and membrane rigidity on red blood cell shape in capillary flow. *Cells.* 12:1529. https://doi.org/10.3390/cells12111529.

Biophysical Journal, Volume *125*

Supplemental information

Impact of red blood cell rigidity on in vivo flow dynamics and lingering in bifurcations

Yazdan Rashidi, Felix Maurer, Selina Wrublewsky, Khadija Larhrissi, Thomas John, Frances B. Gidley, Ashley M. Toye, Lars Kaestner, Christian Wagner, Matthias W. Laschke, and Alexis Darras

Manuscript submitted to **Biophysical** *Journal*
Article

Supplementary Material: Impact of Red Blood Cell Rigidity on in vivo Flow Dynamics and Lingering in Bifurcations

Yazdan Rashidi[1,†,*], Felix Maurer[1,†], Selina Wrublewsky[2,†], Khadija Larhrissi[1,3], Thomas John[1], Frances B. Gidley[4], Ashley M. Toye[4], Lars Kaestner[1,5], Christian Wagner[1,6], Matthias W. Laschke[2,‡], and Alexis Darras[1,7,‡,*]

[1]Experimental Physics, Saarland University, Saarbrücken, Germany
[2]Institute for Clinical and Experimental Surgery, Saarland University, PharmaScienceHub (PSH), 66421 Homburg, Germany
[3]Interdisciplinary Laboratory of Physics (LIPhy), University Grenoble Alpes, 38000 Grenoble, France
[4]School of Biochemistry and Biomedical Sciences, Biomedical Sciences Building, University of Bristol, Bristol, UK
[5]Theoretical Medicine and Biosciences, Saarland University, 66421 Homburg, Germany
[6]Physics and Materials Science Research Unit, University of Luxembourg, L-1511 Luxembourg, Luxembourg
[7]School of Physics, University of Bristol, Tyndall Avenue, Bristol, BS8 1TL, United Kingdom
[*]Correspondence: yazdan.rashidi@uni-saarland.de, alexis.darras@bristol.ac.uk
[†,‡]These author contributed equally to this work.

SUPPLEMENTAL DATA

This PDF file includes: Supporting text, Supp. Fig. S1 to S11, and SI References.

SAMPLE AND ANIMAL PREPARATION

The sample and animal model (mesocricetus auratus) preparations are summarized in Supp. Fig. S1. For the injected suspension, around 2 mL whole blood was extracted from a donor animal. RBCs were separated by centrifugation and divided into two aliquots. One was treated with diamide for rigidification, the other remained untreated. The same washing procedures were used for both cell populations, but different staining protocols were used. CTDR was used for healthy cells, PKH26 for rigid cells, and FITC for plasma. Dorsal skinfold chambers were implanted in pre-weaning animals. After an adjustment time of three days, the freshly prepared stained blood sample was injected and fluorescence microscopy performed immediately afterwards.

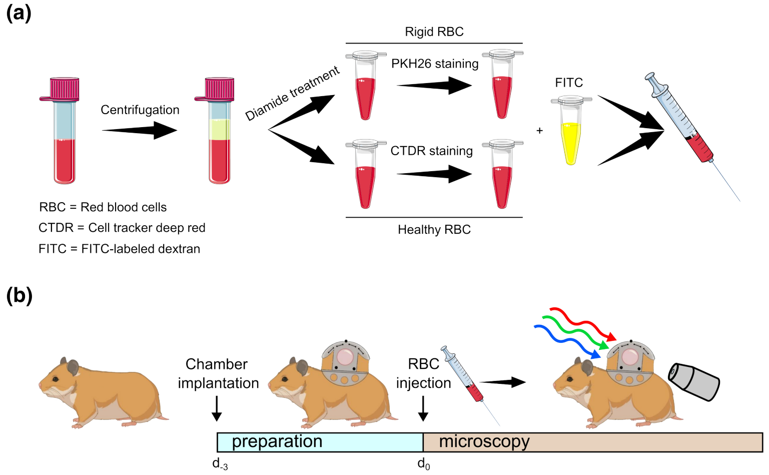

Supplemental Figure **S1**: **Preparation of blood samples and the animal model. (a)** Blood from a donor animal is extracted, RBCs separated and divided into two aliquots. One is treated with diamide for cell rigidification. The two aliquots are stained differently and suspended in FITC-labeled dextran and prepared for injection. **(b)** The skinfold chamber is surgically implanted on d_{-3}. On day of imaging d_0, the blood sample including the three dyes is injected and the chamber is imaged using fluorescence microscopy. Graphics elements adapted from (1).

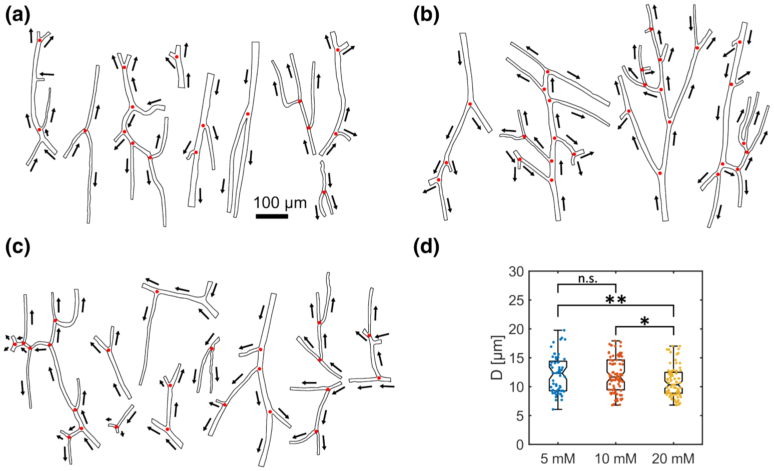

Supplemental Figure **S2**: **Comparison of datasets.** Manually traced masks of the analyzed microvascular geometries are shown for **(a)** 5 mM, **(b)** 10 mM and **(c)** 20 mM diamide. The scaling is equal in those panels. Arrows show the flow direction, red dots mark the analyzed bifurcations. Distributions of the diameter along each vessel are shown in **(d)**. The diameters of 5 mM, $\langle D \rangle = 12.1(4)$ µm, and 10 mM, $\langle D \rangle = 12.0(4)$ µm, are significantly different from the 20 mM dataset, $\langle D \rangle = 10.8(4)$ µm ($p = 0.008$ and $p = 0.013$). The diameters are not significantly different between 5 mM and 10 mM ($p = 0.89$).

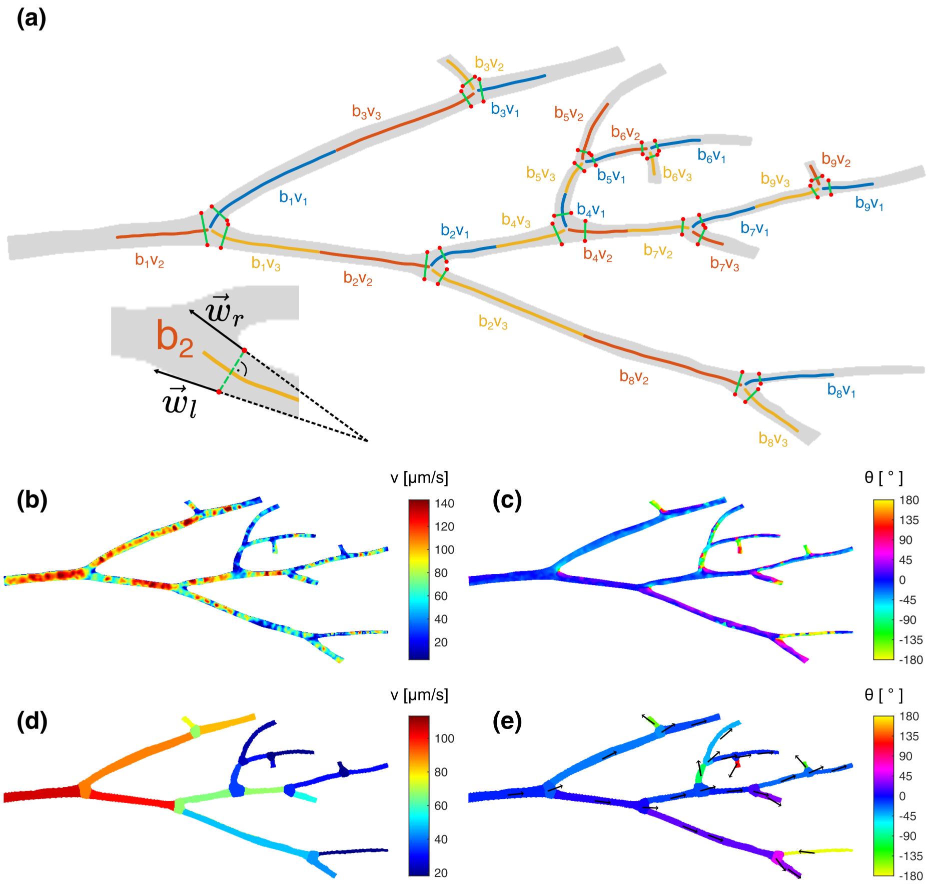

Supplemental Figure **S3**: **Geometry and bulk flow analysis.** **(a)** A mask of the channel interior was created manually, see gray shading. An automatized algorithm found vessel center lines (orange, yellow and blue) and borders of bifurcations (green with red dots). The bifurcation borders were found by the angle between tangential vessel wall vectors $\vec{w}_r$ and $\vec{w}_l$ from two points on the walls where the connection is perpendicular to the vessel center line. Each bifurcation was indexed and has three attached vessels $b_i v_j$, $i = 1, ..., 8$, $j = 1, ..., 3$. **(b)** The Lukas Kanade estimation for optical flow was applied on the footage of stained plasma to estimate the bulk flow speed. **(c)** The flow direction (θ w.r.t. x-axis) was extracted from the flow components. **(d)** Average flow in each region. **(e)** average angle in each region. All bifurcations were arterioles except b_8.

IMAGING AND IMAGE PROCESSING

Microscopic videos were recorded as sequences of $H \times 1024\,\text{px}^2$ grayscale images in uint16 format, where H (≤ 638) depends on the chosen cropped region of interest. The FITC-labeled dextran stained plasma was recorded at 250 fps, CTDR stained RBCs at 50 fps, and PKH26 stained RBCs at 100 fps due to the differing fluorescence signal intensities between stains, while using the maximum allowable exposure time. Each selected region of the vascular network was recorded first with excitation of FITC, afterwards with an alternation between CTDR and PKH26 illumination for a total of five times. In each sequence 2250 frames were recorded. The first two sequences of each cell type were not analyzed to exclude the duration of the tissue adjustment to the illumination. Experiments were carried out for different concentrations of diamide, 5 mM, 10 mM and 20 mM. Supp. Fig. S2 shows a comparison of the three datasets. The stained plasma image is a two-dimensional projection of the three-dimensional vascular network. In a first step, the average plasma image was computed. Vessel walls were identified and traced manually, resulting in a number of single or cascaded bifurcations for analysis. Image processing algorithms performed geometrical measurements on the traced vessel outlines. The numbers of analyzed bifurcations are 18 for 5 mM, 28 for 10 mM and 24 for 20 mM, see Supp. Fig. S2. The distributions of diameters are shown in Supp. Fig. S2. The diameters in the datasets for 5 mM and 10 mM are not significantly different, while both are significantly different from the 20 mM dataset. The 20 mM dataset contains a large portion of smaller capillary vessels, shorter in length and smaller in diameter. Manually drawn masks were input into a homemade algorithm to detect vessel center lines and bifurcation areas, see Supp. Fig. S3 **(a)**. This detection is

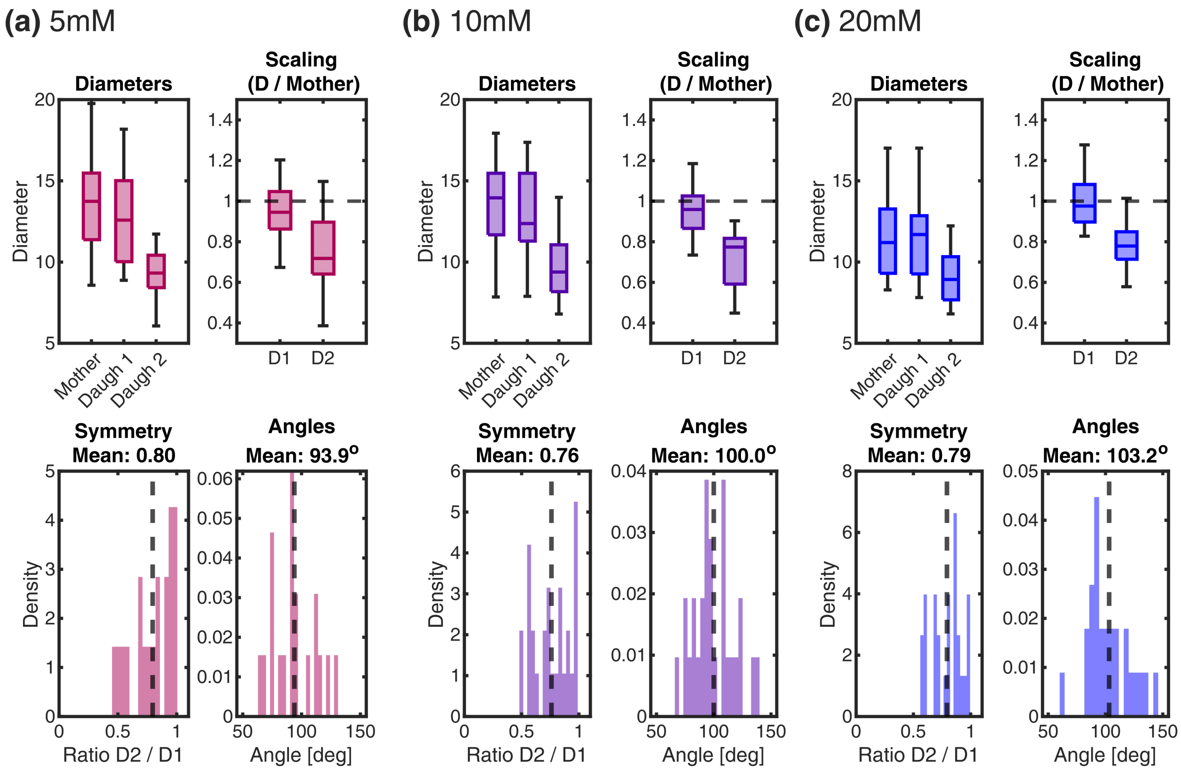

Supplemental Figure **S4**: **Geometry characteristics of the network.** Panels (a–c) display the geometric features of the analyzed network for diamide concentrations of 5 mM, 10 mM, and 20 mM, respectively. For each concentration, four panels are shown: the first panel presents the diameter of each segment at the bifurcations; the second panel shows the fractional diameters of the daughter branches relative to the mother branch, where D1 corresponds to the first daughter branch and D2 to the second; the third panel illustrates the distribution of fractional diameters of the daughter branches; and the fourth panel depicts the distribution of angles between the two daughter branches.

purely based on geometry or morphology. Center lines were detected after skeletonization. The angle of the right vessel wall $\vec{w}_r$ to the left wall $\vec{w}_l$ was used to determine the transition into the bifurcation area by the angle of the vessel walls $\sphericalangle(\vec{w}_l, \vec{w}_r) > 18°$. The apparent vessel width was then measured along the vessel. In order to distinguish between arterioles and venules, we estimated the bulk flow through the network. The Lukas-Kanade algorithm for optical flow estimation was employed to extract flow components from the fluorescence footage of stained plasma, where cells appear dark due to absorption and lack of emission. The bulk flow velocity vectors were extracted, see Supp. Fig. S3 **(b)** and **(c)**. For increased robustness, the average flow in each vessel is computed. This level of resolution was sufficient for subsequent analysis. The average flow and flow direction in each region were used for a predictive search in the cell tracking algorithm.

ADDITIONAL VELOCITY DATA

For the concentrations 5 mM and 20 mM the cell velocities in different regions of the bifurcation are presented in Supp. Fig. S6, while data for 10 mM is shown in the main text. Overall, the data show the same trends compared to 10 mM. At 5 mM compared to 10 mM, smaller differences in deceleration between healthy and rigid cells in the bifurcation area can be observed (Supp. Fig. S6 **(e)**), and no significant difference in acceleration can be observed at the beginning of the daughter branches (Supp. Fig. S6 **(f)**). This might be attributed to the lower concentration of diamide and associated lower rigidity of the rigid cell population. The 20 mM dataset shows a slightly higher speed of rigid cells in the mother branches on the 10 % level of significance, while the remaining trends are comparable to the 10 mM dataset. The higher concentration of diamide should have an equal or stronger effect on cell rigidity, increasing the differences between cell populations. However, the 20 mM dataset contains different vascular geometries compared to the 5 mM and 10 mM datasets, which share a similar set of geometries, see Supp. Fig. S2. Therefore a direct comparison of the 20 mM condition has a reduced interpretive value.

speed differences for 5 mM and 20 mM between bifurcation regions are shown in Supp. Fig. S7. For 5 mM the transitions

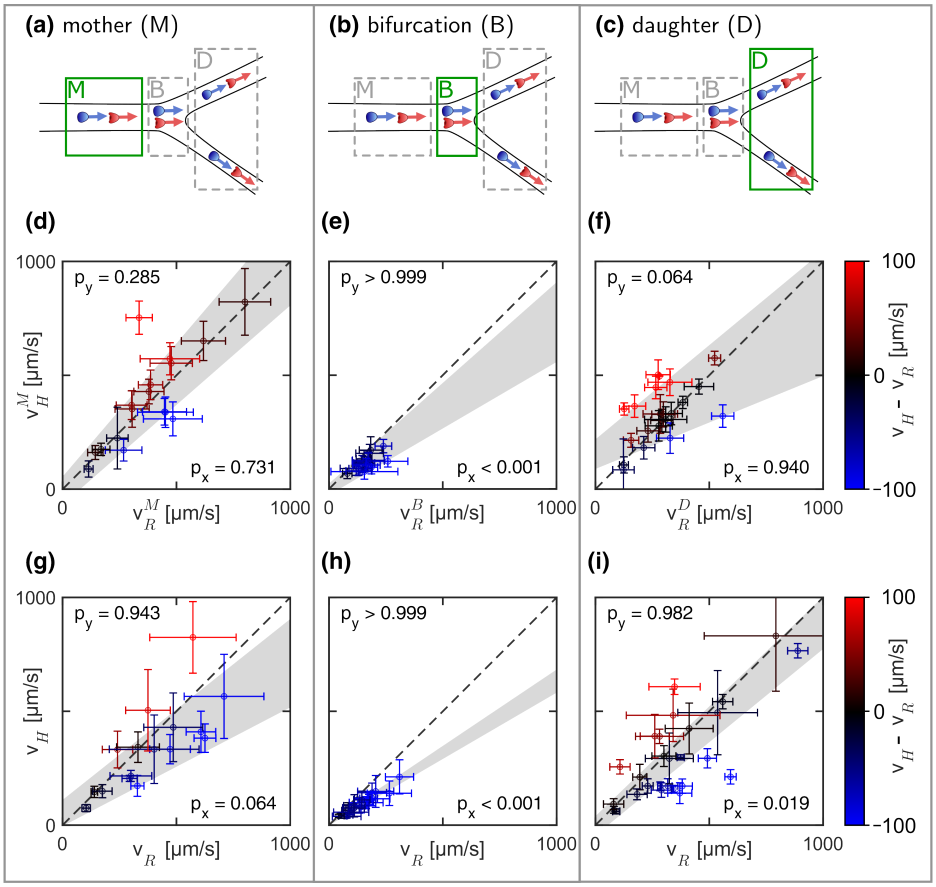

Supplemental Figure **S5**: **Comparison of mean speed for healthy vs. rigid RBCs (5 mM and 20 mM Diamide) across bifurcation regions. (a)-(c)** Schematics of a bifurcation with the ROI as mother vessel (M), bifurcation (B), or daughters (D) highlighted in green, healthy cells in red and rigid cells in blue, and below the mean velocities in each ROI respectively, with rigid RBC velocities on the x-axis and healthy RBC velocities on the y-axis. **(d)-(f)** data for 5 mM, **(g)-(i)** data for 20 mM. Panels share equal y-axes. Each data point shows data from one bifurcation. The dashed line represents equal velocities (identity line). Data points above this line, where healthy RBCs have higher velocities, are shaded increasingly red with greater distance from the line $v_H - v_R$. Points below the line, indicating higher velocities for rigid RBCs, are shaded increasingly blue, see colorbar. Error bars reflect the standard deviation of the cell population. P-values p_x and p_y denote the statistical significance from the sign rank test: $p_y < 0.05$ indicates significantly higher velocities for healthy RBCs, and $p_x < 0.05$ indicates significantly higher velocities for rigid RBCs. The gray filled area shows the 95 % confidence range of a linear regression.

from mother branch to bifurcation and bifurcation to daughter branches exhibit the same behavior as for 10 mM in the main text Fig.4, with a stronger deceleration effect for healthy cells. The 20 mM dataset does not show these clear trends. The difference from comparing average velocities in Supp. Fig. S6 is that the data in Supp. Fig. S7 is correlated, as differences from the transition of individual cells are taken. Specifically in the 20 mM dataset, the velocity data, Supp. Fig. S6 **(g)** indicates that rigid cells are slightly faster in the mother branches. A possible reason might be that vessels in the 20 mM dataset are shorter. The length might not be sufficient for a lateral migration of interacting cells back to the vessel center. We showed that the number of interacting cells is also higher for healthy cells in the 20 mM dataset (Supp. Fig. S8). There are two mechanisms that could potentially increase the fraction of interacting cells and the average lingering time for one cell type over the other. The first is a distribution closer to the apex leading to a higher number of cells that can interact with the apex. The second mechanism is a higher deformability leading to stronger deformation in case of interaction and lingering. For all datasets we can exclude the first mechanism as a reason for the observed differences between healthy and rigid cells. Supp. Fig. S8 **(a)-(c)** show the fraction of cells that can interact with the apex given by the number of trajectories with points located within 4 μm of the apex. There are no significant differences between healthy and rigid conditions for all concentrations of diamide. This means that the interaction probability for healthy and rigid cells by their spatial distribution is similar and differences in the lingering time and

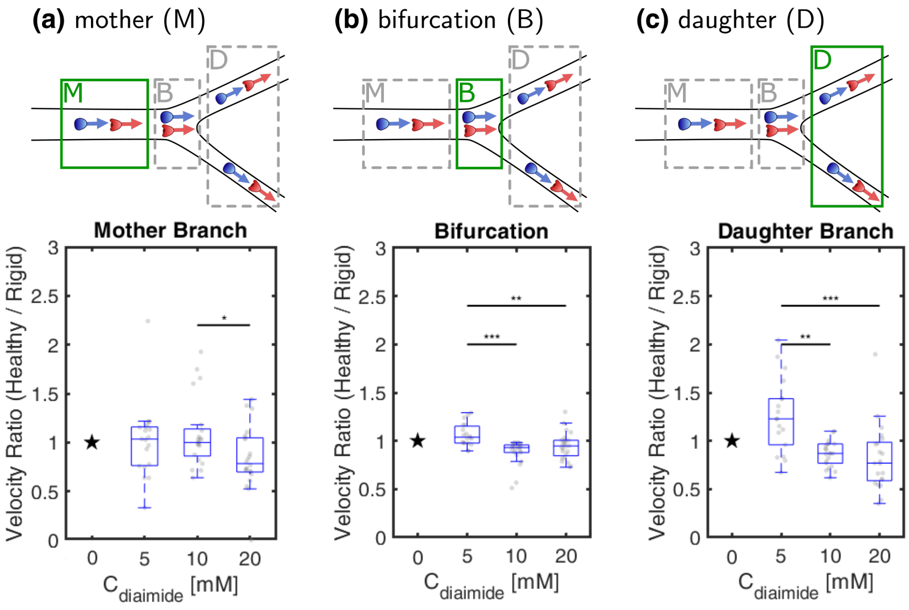

Supplemental Figure **S6**: **Velocity ratio for different diamide concentrations in each segment of the bifurcations. (a)-(c)** Schematics of a bifurcation with the ROI as mother vessel (M), bifurcation (B), or daughters (D) highlighted in green, healthy cells in red and rigid cells in blue, and below the velocities ration as a function of diamide concentration in each ROI respectively. The value at 0 mM represents the expectation, which should be equal to one, as indicated by star. Each data point shows the average for one bifurcation, and statistical significance was evaluated using a two-sample Student t-test. Significant differences are marked with a (*), indicating a p-value less than 0.05.

lateral migration can be attributed solely to differences in deformability. The lingering Peclet number is higher for healthy cells in all datasets, see Supp. Fig. S8 **(d)-(f)**.

LATERAL MIGRATION OF HEALTHY CELLS

The speed distributions of healthy and rigid cells in different regions around the bifurcation are compared in the main text, Fig. 6. In general, the variance of the speed distribution in the beginning of the daughter branch is higher than in the end. Healthy cells exhibit a sub-population of slower-moving cells in the beginning which is reduced in the end of the daughter branch. For the example bifurcation in main Fig. 6**(a)**, the total distance versus the total time spent in each region is shown in Supp. Fig. S9. The two-dimensional distributions show a similar drop shape in each region and for both cell types. There is a tail that appears only in the distribution of healthy cells in the beginning of daughter branches. It belongs to cells that move approximately the same distance as the average distance of all cells but need more time. We conclude that this fraction of cells corresponds to migrating cells interacting with the apex.

To quantify the change in velocity distribution at a bifurcation, we first define the Velocity Drop Ratio (R) for a specific cell type. Let v_M be the velocity of the cell in the mother vessel, and v_{D1} and v_{D2} be the velocities in the first and second daughter vessels, respectively. The partition asymmetry for a given cell type is calculated as the ratio of the velocity drop in the second daughter branch to the velocity drop in the first daughter branch:

$$R = \frac{|v_m - v_{D2}|}{|v_m - v_{D1}|}. \tag{1}$$

In our experiment, we measure this ratio simultaneously for two populations within the same geometry. To determine how cell stiffness alters flow partitioning relative to the healthy baseline, we define the comparative metric δ as the ratio of the healthy drop ratio to the rigid drop ratio:

$$\delta = \frac{R_H}{R_{rigid}} = \frac{\left(\frac{|v_m - v_{D2}|}{|v_m - v_{D1}|}\right)_H}{\left(\frac{|v_m - v_{D2}|}{|v_m - v_{D1}|}\right)_{rigid}}. \tag{2}$$

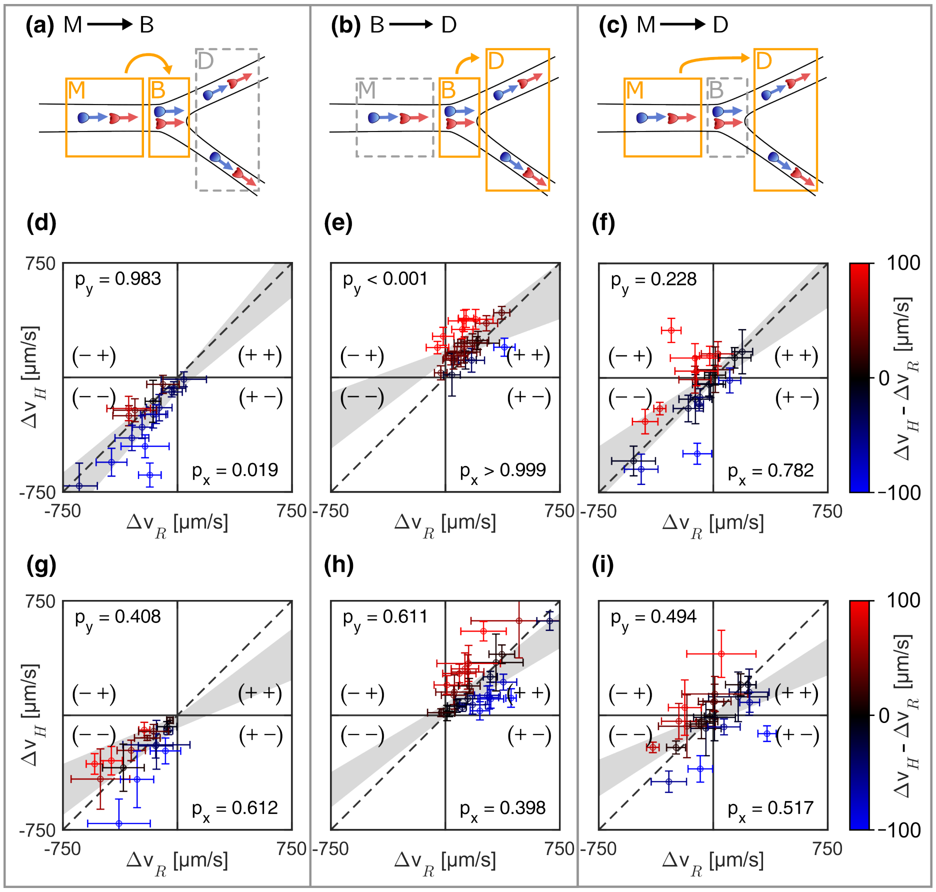

Supplemental Figure **S7**: **Comparison of change in speed for healthy vs. rigid (5 mM and 20 mM Diamide) RBCs across bifurcation regions. (a-c)** Schematics of a bifurcation with the compared regions from mother vessel (M) to bifurcation (B), bifurcation (B) to daughter (D), and mother (M) to daughter (D) highlighted in orange. **(d-f)** Changes in speed, defined as downstream speed minus upstream speed, respectively, rigid RBCs on the x-axis and for healthy RBCs on the y-axis, for 5 mM. **(g-i)** Data for 20 mM diamide concentration. Panels share equal y-axes. The dashed line represents equal velocities (identity line), while solid lines indicate zero change for rigid and healthy RBCs. The point color is according to the distance $\Delta v_H - \Delta v_R$, see colorbar. Error bars reflect statistical experimental uncertainties. A reference to each quadrant by the signs of x and y values is given by (++), (−+), (−−), and (+−). P-values p_x and p_y denote the statistical significance from the sign rank test: $p < 0.05$ indicates significantly higher differences, i.e. a stronger acceleration for (++) or deceleration for (−−). The gray filled area shows the 95 % confidence range of a linear regression.

Supp. Fig. S10 illustrates the distribution of δ across three distinct stiffness conditions (5 mM, 10 mM, and 20 mM). Statistical significance against a null hypothesis of $\delta = 1$ is indicated above each group.

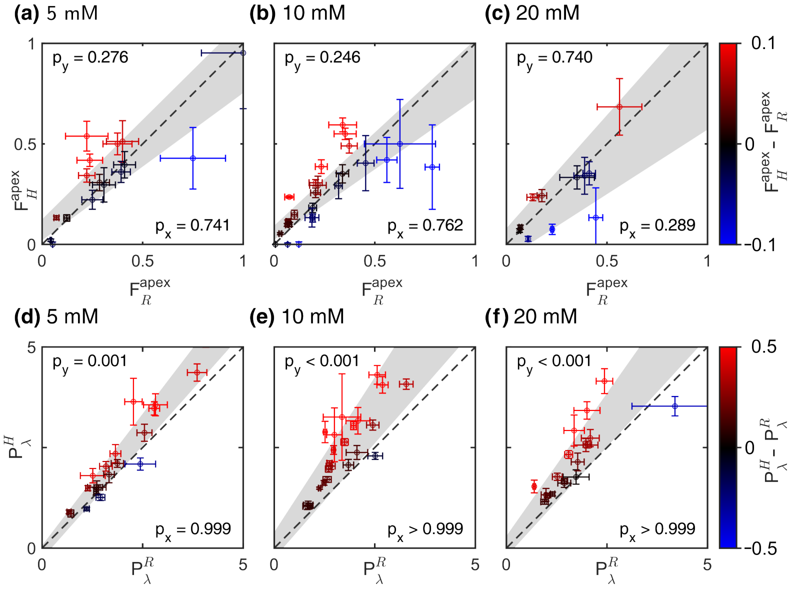

Supplemental Figure **S8**: **Fraction of cells near the apex and lingering. (a-c)** The axes' variables F^{apex} are the number of trajectories with points closer than 4 µm to the apex of the bifurcation divided by the total number of trajectories. Each panel shows the data for a different diamide concentration, **(a)** 5 mM, **(b)** 10 mM, **(c)** 20 mM. **(d-f)** The lingering Peclet number P_λ is the residence time normalized by the advection time. It is significantly higher for healthy cells in all datasets, **(d)** 5 mM, **(e)** 10 mM and **(f)** 20 mM.

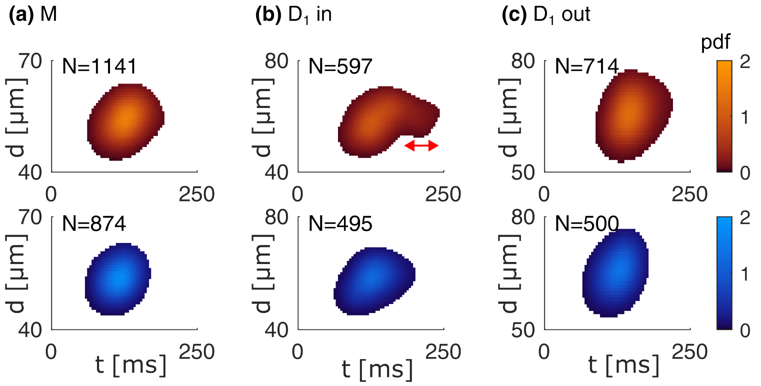

Supplemental Figure **S9**: **Distance versus time in bifurcation regions. Two-dimensional distributions.** Diagrams show the probability density by kernel density estimation of the total distance and the total time spent in **(a)** the end of the mother, **(b)** the beginning of the daughter, and **(c)** the end of the daughter branch. Data for healthy cells in the top row is colored in red, for rigid cells in blue, and the number of detected trajectories *N* is provided. The red double arrow indicates a distribution tail only found for healthy cells in the beginning of the daughter branch. At a similar distance, the tail expands approximately 50 ms in time.

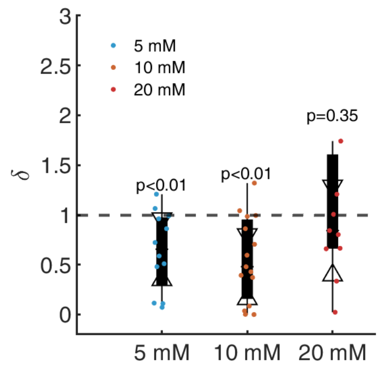

Supplemental Figure **S10**: **Comparison of Flow Asymmetry (δ).** Boxplots showing the ratio of velocity drops for varying stiffening conditions. The horizontal dashed line at $\delta = 1$ represents zero deviation from healthy behavior. (n varies per condition; P-values calculated via t-test against 1).

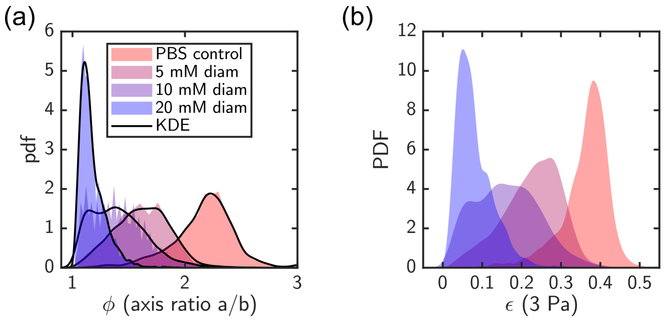

Supplemental Figure **S11**: **Distributions of RBCs elongation under well-controlled shear stresses.** **(a)** Measurements of the elongation, **(b)** associated deformation.

REFERENCES

1. BioRender, 2024. BioRender. https://biorender.com. Accessed: 2025-05-15.
